# The MADD-3 LAMMER Kinase Interacts with a p38 MAP Kinase Pathway to Regulate the Display of the EVA-1 Guidance Receptor in *Caenorhabditis elegans*

**DOI:** 10.1371/journal.pgen.1006010

**Published:** 2016-04-28

**Authors:** Serena A. D’Souza, Luckshi Rajendran, Rachel Bagg, Louis Barbier, Derek M. van Pel, Houtan Moshiri, Peter J. Roy

**Affiliations:** 1 Department of Molecular Genetics, University of Toronto, Toronto, Ontario, Canada; 2 The Donnelly Centre for Cellular and Biomolecular Research, University of Toronto, Toronto, Ontario, Canada; 3 The Collaborative Programme in Developmental Biology, University of Toronto, Toronto, Ontario, Canada; University of California San Francisco, UNITED STATES

## Abstract

The proper display of transmembrane receptors on the leading edge of migrating cells and cell extensions is essential for their response to guidance cues. We previously discovered that MADD-4, which is an ADAMTSL secreted by motor neurons in *Caenorhabditis elegans*, interacts with an UNC-40/EVA-1 co-receptor complex on muscles to attract plasma membrane extensions called muscle arms. In nematodes, the muscle arm termini harbor the post-synaptic elements of the neuromuscular junction. Through a forward genetic screen for mutants with disrupted muscle arm extension, we discovered that a LAMMER kinase, which we call MADD-3, is required for the proper display of the EVA-1 receptor on the muscle’s plasma membrane. Without MADD-3, EVA-1 levels decrease concomitantly with a reduction of the late-endosomal marker RAB-7. Through a genetic suppressor screen, we found that the levels of EVA-1 and RAB-7 can be restored in *madd-3* mutants by eliminating the function of a p38 MAP kinase pathway. We also found that EVA-1 and RAB-7 will accumulate in *madd-3* mutants upon disrupting CUP-5, which is a mucolipin ortholog required for proper lysosome function. Together, our data suggests that the MADD-3 LAMMER kinase antagonizes the p38-mediated endosomal trafficking of EVA-1 to the lysosome. In this way, MADD-3 ensures that sufficient levels of EVA-1 are present to guide muscle arm extension towards the source of the MADD-4 guidance cue.

## Author Summary

In most animals, the physical meeting of the pre- and post-synaptic membranes of the neuromuscular junction occurs via axonal extension towards the muscle. In nematodes, however, motor axons do not extend towards the muscle and instead form a dorsal and ventral cord with relatively few branches. To make the physical connection, the body wall muscles extend membrane projections called muscle arms to the motor axons within the dorsal and ventral cords. Through previous genetic and biochemical analyses with the nematode *C*. *elegans*, we identified a neuronally-expressed muscle arm chemoattractant (MADD-4) and its muscle-expressed co-receptor complex (UNC-40/EVA-1). Here, we report our discovery of *madd-3*, which encodes a LAMMER kinase that is expressed in muscles to regulate muscle arm extension. Genetic analyses revealed that MADD-3 may inhibit a p38 MAP kinase pathway whose normal function is to decrease the abundance of the EVA-1 receptor. In the presence of MADD-3, the activity of the p38 pathway is relatively low, and EVA-1 levels are consequently relatively high. Without MADD-3, the p38 pathway is freed to decrease the abundance of EVA-1. The relationships that we have uncovered between MADD-3, the p38 Map Kinase pathway, and the EVA-1 receptor provide one explanation for the muscle arm defects observed in *madd-3* mutants.

## Introduction

**Fig 1:**
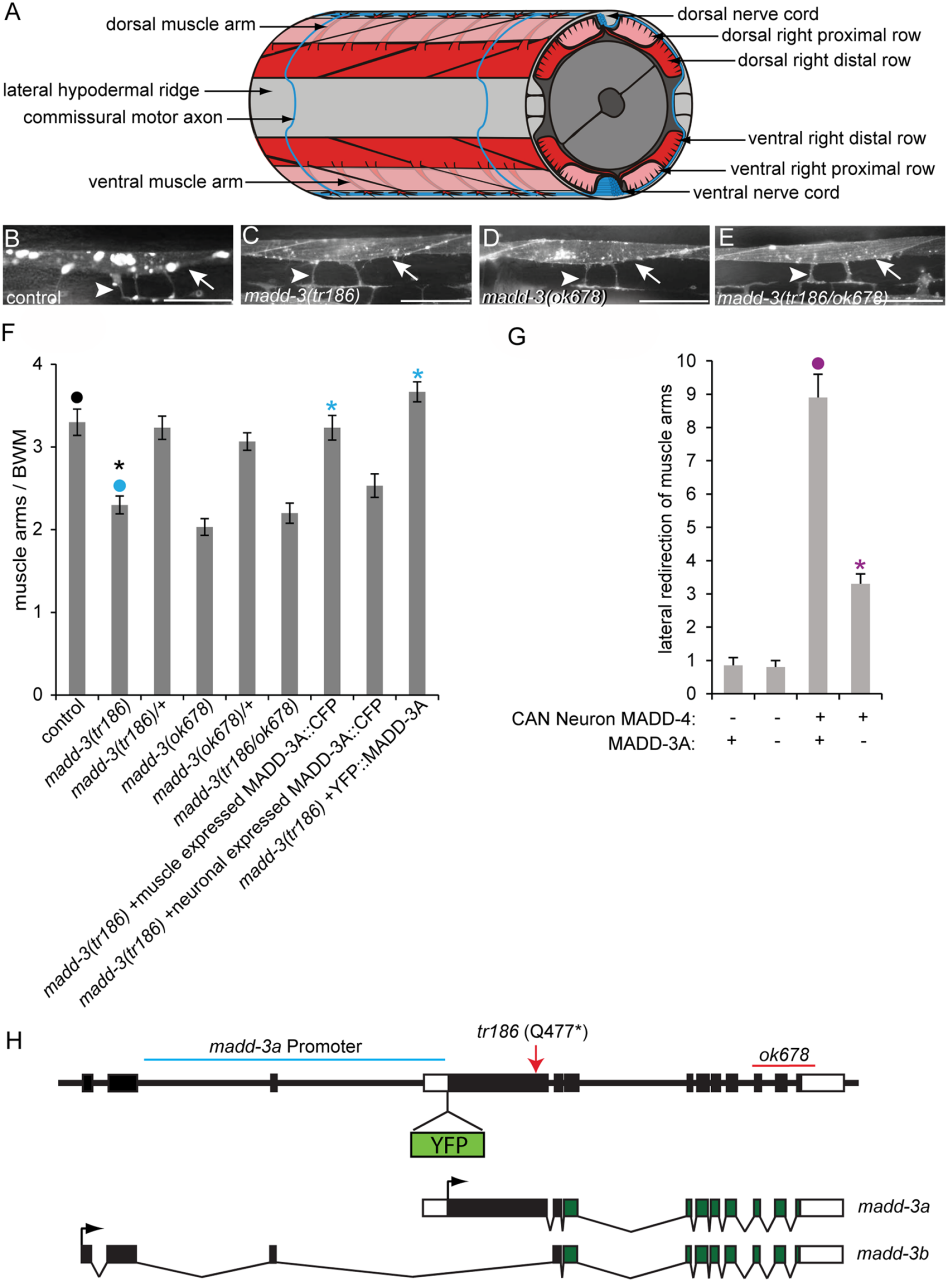
The muscle arm extension defects of *madd-3* mutants. **A.** A schematic of muscle arms and their motor neuron targets. **B-E.** The muscle arms of animals of the indicated genotype. All animals harbour the *trIs30* transgene. Scale bars are 25μm. **F.** The number of muscle arms that extend from muscle VL11 towards the ventral nerve cord. **G.** The number of muscle arms that extend towards MADD-4 that is ectopically expressed by the lateral CAN neuron. Numbers shown are for the left side of the animal. The right side behaves similarly. In both graphs an asterisks indicates a significant difference (p<0.01) compared to the data point indicated with a closed circle with the same color as the asterisks. **H.** Schematics of the genomic *madd-3* locus and resulting transcripts. The position of the *tr186* allele and the *ok678* deletion are indicated. The position where a YFP tag was inserted in the context of the fosmid reporter (see text) is shown. The region of DNA used to drive the madd-3a YFP transcriptional reporter is indicated with a blue line. Black boxes indicate exons and white boxes are untranslated regions.

The guided migration of cells and their extensions during development is essential to the proper execution of the body plan. In nematodes, the body wall muscles extend membrane protrusions called muscle arms to the motor axons where they form a neuromuscular junction (Fig 1a) [1]. Using the nematode *Caenorhabditis elegans* as a model system, we have identified several components that guide the muscle arms to their target [2,3,4]. These components not only guide muscle arms, but also play a role in guiding other cell extensions in *C*. *elegans*, including axons. Although other phyla exhibit a variety of forms of muscle membrane extensions [5,6], muscle arms are likely a nematode-specific adaptation. Despite this, the components that we have found to guide muscle arms are evolutionarily conserved. Some are known to play guidance roles in more complex systems [2], while the role of others remain to be determined.

Through forward genetic screens, we identified a protein called MADD-4 that is secreted by motor neurons and attracts muscle arms [4]. The *madd-4* gene was the fourth complementation group identified in a screen for mutant genes that confer a Muscle Arm Development Defective (MADD) phenotype [2,4]. MADD-4 is ‘a disintegrin and metalloproteinase with thromobospondin motif like (ADAMTSL)’ family member and lacks the enzymatic domains of true ADAMTS proteins [1]. MADD-4 interacts with a muscle-expressed UNC-40/EVA-1 co-receptor complex to elicit muscle arm extension [7]. EVA-1, a single-pass transmembrane protein with two galactose-binding domains, interacts strongly with MADD-4. EVA-1’s primary role in the co-receptor complex is likely to facilitate MADD-4’s interaction with the UNC-40 receptor, which is an ortholog of the mammalian DCC receptor. In the absence of EVA-1, the UNC-6 netrin ligand antagonizes MADD-4’s interaction with UNC-40 [7]. We also discovered a tripartite motif protein called MADD-2 that serves as an adaptor protein to facilitate an interaction between UNC-40 and UNC-73, a Rho GEF [3]. In addition to a variety of downstream cytoskeletal modulators, we found that select components of the sarcomere’s thick filaments and components required for sarcomere anchorage are necessary for proper muscle arm extension for reasons that are less clear [2]. Genetic analyses indicate that an UNC-6-UNC-40-MADD-2 pathway functions in parallel to a MADD-4-UNC-40-EVA-1 pathway to guide ventral muscle arms to the ventral cord [7]. Given that MADD-2 and MADD-4 are conserved proteins that had not been previously recognized to play a role in guided migration in any system, the study of muscle arm extension has been a fruitful avenue to identify new guidance components.

Here, we describe the third novel complementation group, called *madd-3*, that we identified in our screens for genes required for muscle arm extension. MADD-3 is a member of the LAMMER dual-specificity protein kinase family that is conserved throughout eukaryotes. The LAMMERs are named after a well-conserved EHLAMMERILG motif in subdomain X of the kinase domain [8]. A well-characterized function of LAMMER kinases is the phosphorylation of SR proteins, which regulate pre-mRNA splicing, alternative splicing, and additional post-splicing activities [9,10,11]. In mammals, the LAMMER kinase CLK1 phosphorylates SR proteins, which in turn, regulate the alternative splicing of protein kinase C βII (PKCβII) [12]. *Doa* (Darkener of Apricot) encodes the sole LAMMER kinase in *Drosophila* and has multiple isoforms with distinct sub-cellular distributions [13,14]. The 55 kDa Doa isoform regulates the splicing of Doublesex (*dsx*) mRNA, which is a key regulator of sex-determination in flies, via the phosphorylation of SR proteins.

An increasing number of non SR-proteins are being identified as *in vivo* substrates of the LAMMERs. For example, the fission yeast *S*. *pombe* LAMMER kinase Lkh1 phosphorylates RNA binding protein Csx1 and the transcription repressors TUP11 and TUP12 [15]. In mouse hepatocytes, the CLK2 LAMMER kinase represses the activity of PGC-1α, a transcriptional coactivator, by phosphorylating its RS domain [16].

LAMMER kinase activity extends beyond the nucleus. For example, the 105 kDa Doa isoform is localized exclusively in the cytoplasm and unlike the nuclear-localized 55kDa isoform, does not appear to affect alternative splicing [14,17,18,19]. The 225 kDa Doa isoform binds microtubules and phosphorylates the microtubule binding protein EF1γ, which in turn, negatively regulates the transport of organelles in *Drosophila* S2 cells [20]. This is the first clear role for a LAMMER kinase family member in the cytoplasm.

We report that the *C*. *elegans* LAMMER kinase MADD-3A is enriched in the cytoplasm and promotes muscle arm extension by, at least in part, ensuring that sufficient levels of EVA-1 are displayed on the plasma membrane. We also found that *madd-3a* mutants exhibit a synthetic lethal phenotype when combined with loss-of-function mutations in *unc-54*, which encodes the myosin heavy chain MHC B [21]. Through a second forward genetic screen for suppressors of the synthetic lethality, we found that loss-of-function of the p38 MAP kinase pathway suppresses the mutant phenotypes of *madd-3a*. Further analyses suggest that the p38 pathway promotes the lysosomal degradation of EVA-1 and that MADD-3A ensures sufficient EVA-1 levels by negatively regulating the p38 pathway. This is the first demonstrated link between a LAMMER kinase and a MAP kinase pathway.

## Results

### *madd-3* Functions Cell-Autonomously to Regulate Muscle Arm Extension

To find new components required for muscle arm extension, we carried out a forward genetic screen that has been previously described [4]. Briefly, we used the RP112 strain that expresses membrane-anchored YFP in the distal body wall muscles to enable epifluorescent visualization of muscle arms. We randomly mutagenized RP112 parents with ethyl methanesulfonate and screened their F2 grand-progeny for mutants with reduced muscle arm numbers. We isolated 15 mutants with muscle arm defects from a total of 20 000 mutant haploid genomes. From these mutants, we identified four alleles of *madd-2*, three alleles of *unc-40*, three alleles of *madd-4*, and one allele (*tr186*) of a new complementation group that we call *madd-3*. The muscle arm defects of *madd-3(tr186)* (Fig 1b–1e) are less severe than those caused by nulls in the aforementioned genes[4].

We previously developed an assay to test whether a gene that is required for muscle arm extension is also required for extension towards the secreted muscle arm attractant MADD-4 [4]. When expressed from the lateral CAN neuron, MADD-4 redirects muscle arm extension laterally [4]. Loss-of-function mutations in *unc-40*, *madd-2*, and *eva-1* suppress these redirected muscle arms [4]. We found that the *madd-3(tr186)* mutation also suppresses muscle arm extension towards ectopic MADD-4 (Fig 1g), suggesting that it too is required in the attractive response.

We used molecular techniques to map *madd-3(tr186)* to linkage group X between map positions 16.66–16.68, which defines a 135 kb region and includes 26 predicted protein-coding genes (S1 Fig). From the RP1313 strain that harbors *madd-3(tr186)*, we sequenced candidate genes in this region and found that *E02H4*.*3* encodes a nonsense mutation that is specific to the ‘A’ isoform of the locus and leaves the ‘B’ isoform intact (Fig 1h). E02H4.3 encodes the sole LAMMER kinase in the *C*. *elegans* genome (S2 Fig).

We further investigated whether the *E02H4*.*3* mutation is responsible for the muscle arm defects seen in RP1313. First, we found that a publically available allele of *E02H4*.*3 (ok678)*, which is a deletion that affects both *E02H4*.*3* isoforms, has muscle arm defects as severe as *tr186* and fails to complement *tr186* (Fig 1d, 1e and 1f). The severity of *tr186*’s muscle arm defects is not increased when *in trans* over *ok678*, indicating that *tr186* is genetically null for the locus’s role in muscle arm extension. Of note, *ok678* homozygotes are sterile, suggesting that the *madd-3b* isoform has roles beyond muscle arm extension. Second, a transgene that drives the expression of *E02H4*.*3*’s ‘A’ isoform specifically in muscles rescues *tr186*’s muscle arm defects (Fig 1f). By contrast, the expression of *E02H4*.*3*A in neurons failed to rescue *tr186*’s muscle arm defects. These results indicate that *E02H4*.*3* is *madd-3*, and that the ‘A’ isoform functions cell-autonomously in muscles to regulate muscle arm extension. The *C*. *elegans* nomenclature committee has consequently given *E02H4*.*3* the ‘*madd-3*’ trivial name.

**Fig 2:**
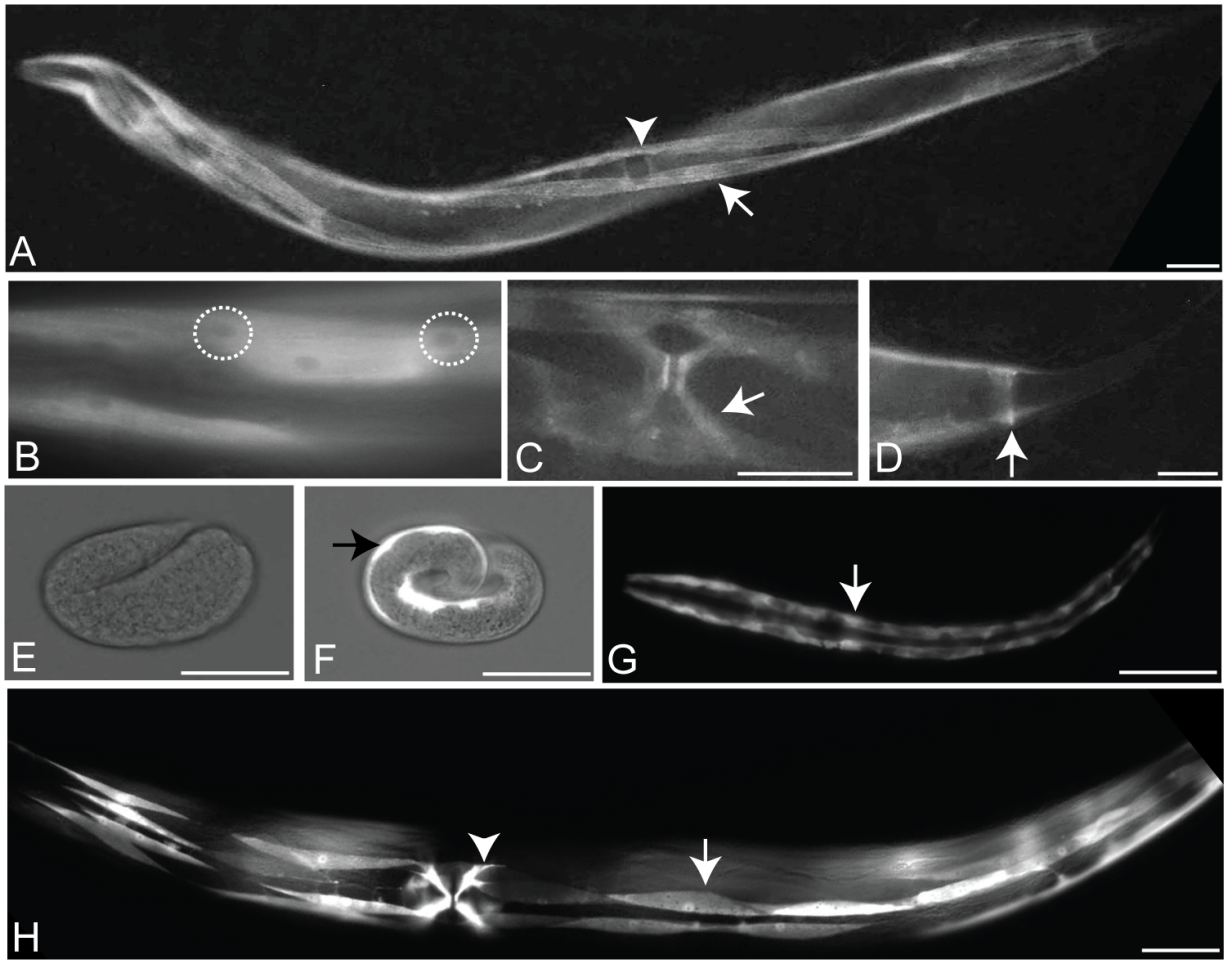
The MADD-3 expression pattern. **A-D.** Expression of a MADD-3A translational reporter (the placement of the YFP tag is shown in Fig 1H). **A.** A young adult worm. An arrow indicates a body wall muscle within one of the ventral quadrants, the arrowhead indicates the position of the vulval slit. The animal is twisted due to a marker within the extra-chromosomal array that carries the reporter. **B.** Body wall muscles showing relatively less YFP in the nucleus (two nuclei are encircled). **C.** The vulva muscles (arrow). **D.** The anal depressor muscle (arrow). **E-H**. Expression of a *madd-3Ap*::YFP transcriptional reporter. **E.** A partial bright-field photograph showing that YFP is not expressed from the *madd-3A* promoter at the embryonic two fold stage. **F.** At the embryonic pretzel stage, YFP expression driven by the *madd-3A* promoter is evident in the body wall muscles (arrow). **G.** An L1 larva with the two rows of body wall muscles showing. **H.** A young adult worm. A single body wall muscle is indicated with an arrow and a vulva muscle is indicated with an arrowhead. Scale bars represent 25 μm.

**Fig 3:**
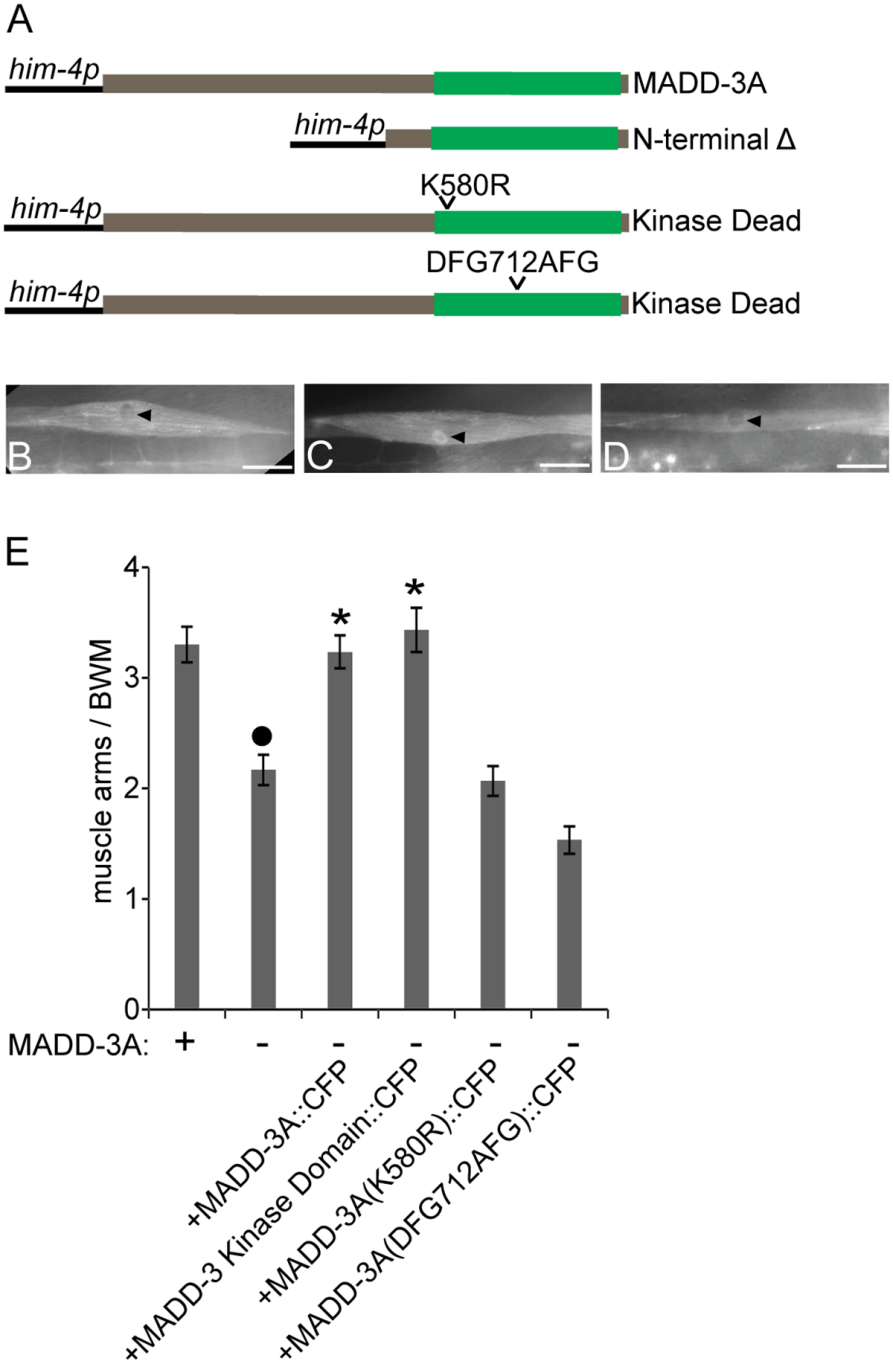
Muscle-specific expression of MADD-3A and its ability to rescue *madd-3(tr186)*’s muscle arm extension defects. **A.** Schematics of a muscle-specific promoter (*him-4p*) driving the expression of wild type and mutant versions of MADD-3A. The kinase domain is schematized in green. All proteins are C-terminally tagged with FLAG and CFP. The construct names are pPRSAD426 (MADD-3A); pPRSAD427 (N-terminal deleted); pPRSAD534 (K580R kinase dead); pPRSAD535 (DFG712AFG kinase dead). **B.** The expression of wild type MADD-3A in a single body wall muscle. The arrowhead indicates lower abundance of MADD-3A from the nucleus. **C.** The expression of MADD-3A without its N-terminal half results in the relative accumulation of the tagged protein in the nucleus (arrowhead). **D.** The K580R kinase dead version of MADD-3A is localized similarly to wild type MADD-3A. The scale bar in B-D represent 25 μm. **E.** The ability of the indicated muscle-expressed proteins to rescue ventral the muscle arm extension defects of *madd-3(tr186)* muscle VL11 is shown (n = 30). The presence of a wild type or *tr186* allele of MADD-3A is indicated with a respective (+) or (-) below the bars of the graph. An asterisks indicates a significant difference (p<0.01) relative to the *madd-3(tr186)* control.

We investigated *madd-3a*’s spatial expression pattern in two ways. First, we recombineered a 29 kb fosmid clone that contains *madd-3* to express an N-terminally YFP-tagged version of MADD-3A (Fig 1h). This fosmid reporter includes sequence 8.3 kb upstream of *madd-3*’s initiator codon and 8.8 kb downstream of *madd-3*’s stop codon, and fully rescues the muscle arm defects of *madd-3(tr186)* (Fig 1f). We detected YFP signal in animals transgenic for the fosmid reporter in only the body wall muscles, vulva muscles and the anal depressor muscle in young larvae and all subsequent stages (Fig 2a–2d). In these animals, as well as those that express a functional CFP-tagged MADD-3A specifically in body wall muscles, the tagged protein appears enriched in the cytoplasm relative to the nucleus (Figs 2b and 3b). Second, we built a transcriptional reporter using 5 kb of sequence upstream of *madd-3a* to drive YFP expression (see Fig 1h). We observed the identical expression pattern with the transcriptional reporter compared to the fosmid reporter, except that body wall muscle expression in embryos was also apparent (Fig 2e and 2f). Consistent with our observation that MADD-3A functions cell-autonomously, we conclude that *madd-3a* is likely expressed in body wall muscles.

### The LAMMER Kinase Domain Is Necessary and Sufficient for MADD-3A’s Regulation of Muscle Arm Extension

MADD-3A is predicted to consist of an N-terminal region of 550 residues that has no recognizable domains and a C-terminal LAMMER kinase domain (Fig 3a). We investigated the role of these two halves on MADD-3A’s function and localization by generating CFP-tagged versions of MADD-3A with either of these two regions mutated. MADD-3A lacking its N-terminal region allows MADD-3A to accumulate in the nucleus in addition to the cytoplasm (Fig 3a, 3b and 3c), suggesting that the N-terminal half may regulate the nuclear-cytoplasmic distribution of MADD-3A. MADD-3A lacking this N-terminal region is sufficient to rescue the muscle arm defects of *madd-3(tr186)* suggesting that the N-terminal region is dispensable for MADD-3A’s regulation of muscle arm extension (Fig 3e). We created two kinase-dead mutations through site-directed mutagenesis (Fig 3a). Neither construct altered MADD-3A’s subcellular localization (Fig 3d), and neither was able to rescue *madd-3(tr186)*’s muscle arm extension defects (Fig 3e). These results indicate that MADD-3A’s kinase domain may be necessary and sufficient to regulate muscle arm extension.

### MADD-3A’s Function Is Not Restricted to a MADD-2 or MADD-4/EVA-1 Pathway

**Fig 4:**
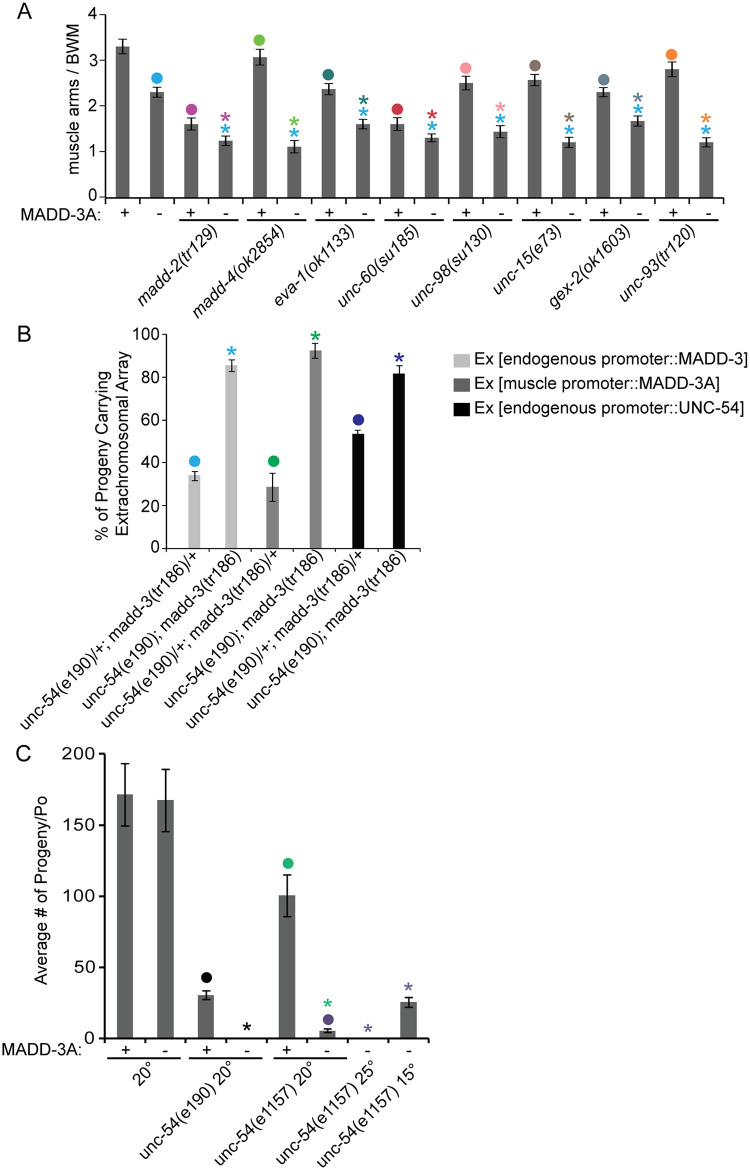
Genetic interaction analyses reveals a synthetic lethal interaction between *madd-3* and *unc-54*. **A.** Double mutant analyses reveals that *madd-3(tr186)* enhances the muscle arm defects of all mutants shown. Counts for muscle VL11 are shown (n = 30). **B.** The viability of *unc-54(e190); madd-3(tr186)* double mutants is dependent on a rescuing array that expresses either MADD-3 or UNC-54. Shown for each genotype are the fraction of F1 progeny that carry the indicated extra-chromosomal (Ex) array from parents that also carried the same array. All double homozygotes are dependent on the array to become viable fertile adults (see text), while double heterozygotes do not. The array carrying the endogenous promoter expresses YFP::MADD-3A within the context of a 29 kb fosmid clone (construct pPRSAD539- see methods); the *myo-3* promoter was used to express MADD-3A specifically in muscles (construct pPRSAD499); UNC-54 was expressed in the muscles from construct pPD5.41 (a gift from Andrew Fire). **C**. *madd-3(tr186)* is synthetic lethal with a temperature sensitive allele of *unc-54(e1157)*. Strains were grown at the indicated temperature (Celsius) for at least 3 days before individual L4s were cloned on to separate plates (n = 8). Progeny of the cloned animals were counted when they reached L4. For all graphs shown, an asterisk indicates a significant difference (*p*<0.05) with respect to the data point indicated with a closed circle of the same color as the asterisks.

In an effort to identify the pathway in which MADD-3A may be working to regulate muscle arm extension, we investigated *madd-3* genetic interactions with other genes known to play a role in muscle arm extension. Double mutant analyses previously revealed that the activity of the EVA-1 transmembrane receptor in directing muscle arm extension is restricted to a MADD-4 pathway because the muscle arm defects of the *eva-1 madd-4* double null mutant are no more severe than either single mutant [7]. We used the same approach to investigate *madd-3(tr186)* and found that it enhanced the muscle arm defects of null mutations in components required for muscle arm guidance (*madd-2*, *madd-4*, *eva-1*) and other components required for extension (*gex-2*, *unc-15*, *unc-60*, *unc-93*, *unc-98*) (*p*<0.05) (Fig 4a). This suggests that MADD-3A activity is not restricted to any single pathway that requires these components.

As part of our systematic investigation, we made the surprising discovery that *madd-3(tr186)* is synthetically lethal with an *unc-54(e190)* null mutation, and the homozygous double mutant could only be maintained in the presence of a transgene that expresses either MADD-3A or UNC-54 in muscles (Fig 4b). We also observed the synthetic lethality when *madd-3(tr186)* was made double with a temperature sensitive allele of *unc-54(e1157)* (Fig 4c). UNC-54 is the sole myosin heavy chain B (MHC B) in the body wall muscles [22,23]. Although severe disruption of muscle development is lethal, *unc-54* null mutants are viable because MHC A is sufficient for contraction. It is currently unclear why *madd-3(tr186)* is synthetic lethal with *unc-54(e190)*.

### Loss-of-Function Mutations in a p38 MAP Kinase Pathway Suppress *madd-3a*’s Synthetic Lethal Interaction with *unc-54*

To identify components that might act downstream of MADD-3A, we carried out a forward genetic screen for mutants that could suppress *madd-3(tr186)*’s synthetic lethal interaction with the *unc-54(e190)* deletion allele. We used a strain called RP2361 that harbors the *madd-3(tr186); unc-54(e190)* double mutation in the background of a rescuing extra-chromosomal transgenic array that expresses a CFP marker and muscle-expressed UNC-54 (S3 Fig). RP2361 also contained a marker (*trIs30*) to allow the visualization of muscle arms. Because of the synthetic lethality, the strain is dependent on the extra-chromosomal array for viability. All healthy RP2361 animals have the array and fluoresce blue. Animals that have lost the extra-chromosomal array either die or are sick and do not yield progeny. The RP2361 animals with the extra-chromosomal array move well because the paralyzed phenotype of *unc-54(e190)* is rescued by the transgenic array.

**Fig 5:**
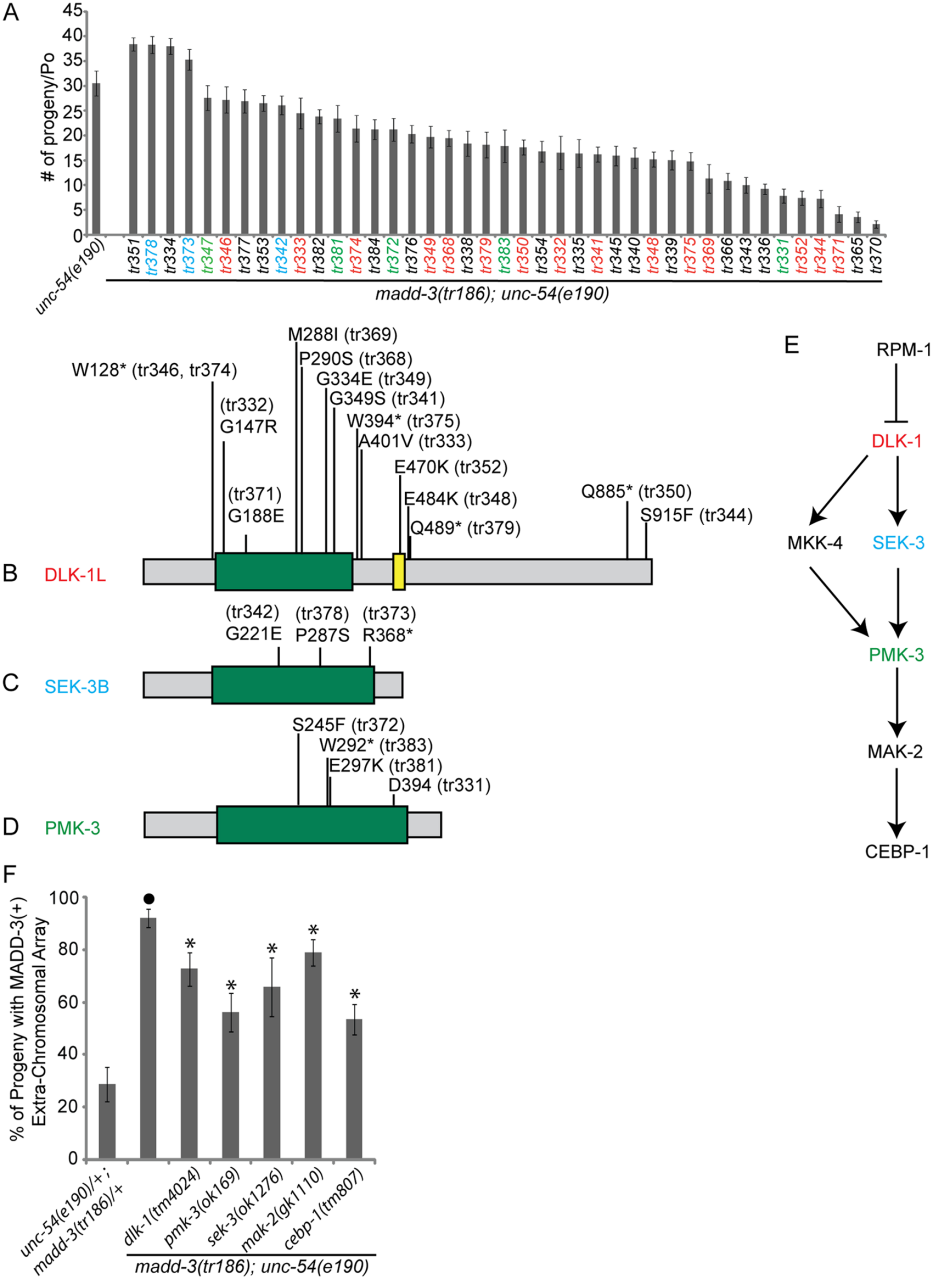
Mutations in the p38 MAP kinase pathway suppress the synthetic lethality of the *unc-54; madd-3* double mutant. **A.** The 41 suppressors that we isolated allow the *unc-54(e190); madd-3(tr186)* double mutants to survive without a rescuing extra-chromosomal array. The average number of progeny thrown by a single adult of the indicated genotype is shown (n = 10). The color of the name of the allele is matched to the color of the MAP kinase components in B-E. **B-D.** Schematics of the indicated MAP kinase pathway components and the position of the mutations that we identified. The green box indicates the kinase domain. **E.** A schematic of the MAP kinase pathway. **F.** Deletion alleles of the p38 MAP kinase pathway suppress the synthetic lethality of the *unc-54; madd-3* double mutant. Shown are the fraction of progeny from individual *unc-54(e190); madd-3(tr186)* double mutants that harbour an extra-chromosomal array that expresses MADD-3A specifically in muscles (from the construct pPRSAD499). The genetic background is indicated. Note that all triple mutant strains survive and propagate without the presence of the extra-chromosomal array. An asterisk indicates a significant difference (*p*<0.05) compared to the data point indicated with a closed circle of the same color as the asterisks.

To identify *madd-3(tr186)* suppressors, we randomly mutagenized RP2361, screened ~240,000 genomes, and isolated 41 viable healthy F2 mutants that no longer had the extra-chromosomal array (Fig 5a). Sequence analyses revealed that none of the suppressors are reversions of the *madd-3(tr186)* point mutations. In principle, this screen could yield suppressors of *madd-3* or *unc-54*. However, all 41 suppressors that we isolated retained the paralysis conferred by the *unc-54(e190)* mutation, indicating that these mutants are likely suppressors of *madd-3(tr186)*.

To identify the mutant genes responsible for the suppression, we reasoned that at least some of these mutant genes might be represented by multiple alleles within our collection and could therefore be identified through whole-genome sequencing of multiple suppressor strains. We sequenced the genome of 25 of the suppressors that are indicated in Fig 5a (with coloured notation) and searched for commonly mutated genes with non-synonymous mutations. Across the 25 sequenced genomes, 1603 protein-coding genes have one unique mutation, 68 genes have unique mutations in two distinct strains, four genes have unique mutations in three distinct strains, one gene has unique mutations in five distinct strains, and one gene has unique mutations in 15 distinct strains (S1 Dataset). Inspecting these mutant genes revealed that we isolated 15 alleles of the Mitogen Activated Protein Kinase (MAPKKK) *dlk-1* (five of which are nonsense), three alleles of the MAPKK *sek-3* (one of which is nonsense), and five alleles of the p38 MAPK *pmk-3* (one of which is nonsense and another is a splicing acceptor mutation) (Fig 5b, 5c and 5d).

An RPM-1 (E3 ubiquitin ligase), DLK-1 (MAPKKK), MKK-4 (MAPKK), PMK-3 (MAPK), MAK-2 (MAPKAP), and CEBP-1 (C/EBP bZip factor) pathway has been previously shown to regulate receptor clustering at presynaptic sites on GABAergic motor neurons in *C*. *elegans* [24,25] (Fig 5e). Hypomorphic mutations in RPM-1 result in abnormal clustering of presynaptic markers [26,27], which is a phenotype that is suppressed by loss-of-function mutations in the aforementioned MAP kinase pathway components [24,25]. Similarly, mutations in RPM-1 lead to defects in the accumulation of the GLR-1 AMPA-type glutamate receptor in interneurons, and mutations in DLK-1 and PMK-3 suppress these defects [28]. In both motor- and interneurons, RPM-1 negatively regulates the DLK-1 pathway by targeting DLK-1 for proteolysis [24,28].

Four lines of evidence indicate that the mutations in the *dlk-1*/*sek-3*/*pmk-3* MAP kinase components are responsible for the observed suppression. First, each of the 23 *dlk-1*, *sek-3*, and *pmk-3* alleles are found in distinct suppressor strains. If these mutations were not related to the observed suppression, some of the *pmk-3* alleles might have been present in strains that also carry a mutation in *dlk-1* for example. Second, a statistical analysis indicates that the chances of identifying 15 alleles of single gene within a collection of 25 strains by random chance alone is zero (S1 Table). Third, non-synonymous mutations in *dlk-1*, *sek-3*, and *pmk-3* were not found upon whole-genome sequencing of 33 strains that were mutagenized in a similar manner in an unrelated project in the lab [29]. Finally, we found that publically-available deletion alleles of *dlk-1(tm4024)*, *sek-3(ok1276)*, *pmk-3(ok169)*, *mak-2(gk1110)*, and *cebp-1(tm807)* are able to suppress the synthetic lethality of the *unc-54; madd-3* double mutant (Fig 5f). We are able to maintain the triple mutants without a rescuing extra-chromosomal array and the animals are healthy, but have *unc-54*-like paralysis, indicating that these mutants suppress *madd-3(tr186)* and not *unc-54(e190)*. As expected, the frequency at which we recovered alleles of the aforementioned MAPK components in the suppressor screen roughly correlates to the length of their coding sequence (see Fig 5).

### MADD-3A Interacts with a p38 MAP Kinase Pathway to Regulate Muscle Arm Extension

**Fig 6:**
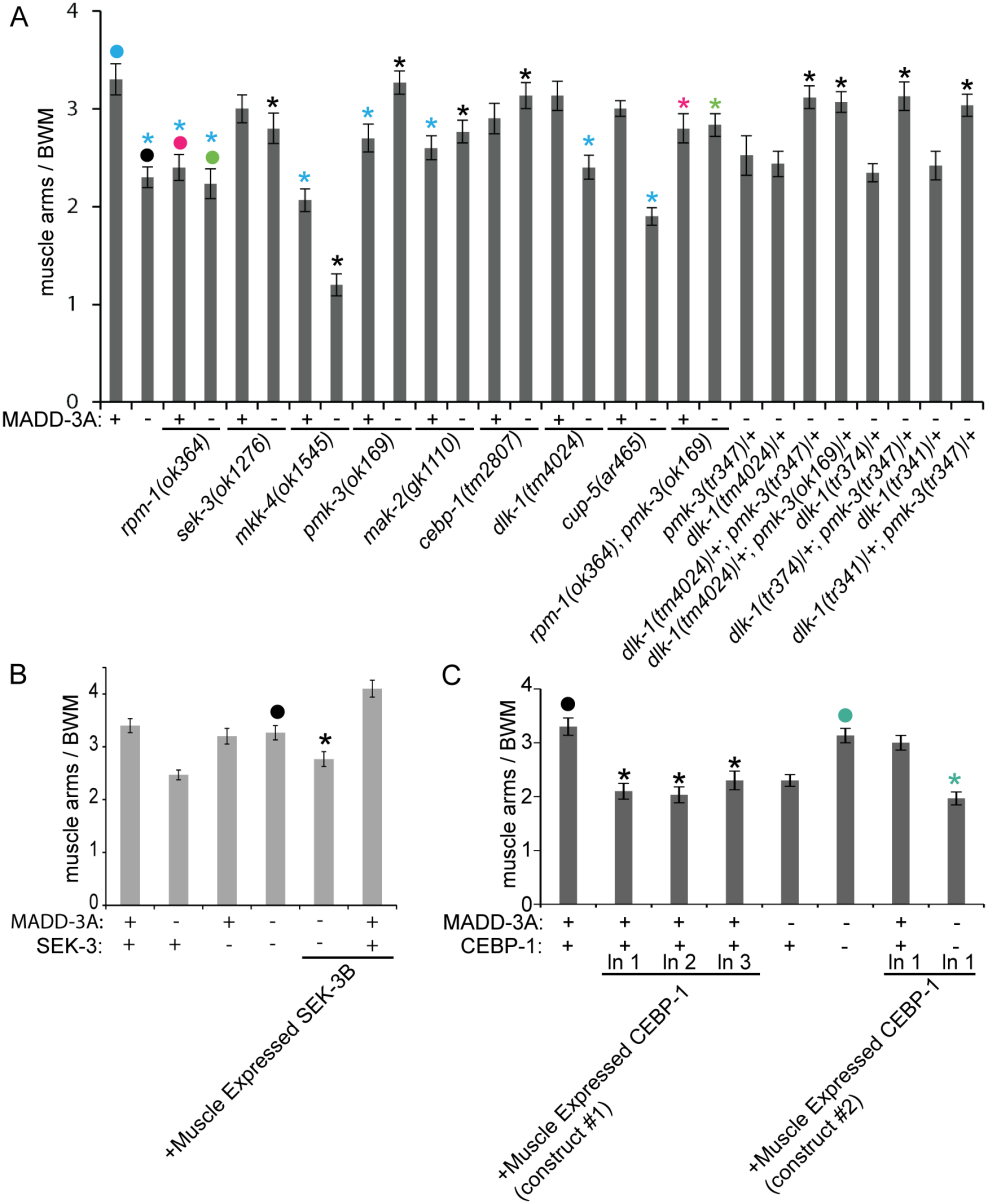
Deletion alleles of MAP kinase pathway components suppress the muscle arm extension defects of *madd-3* mutants. **A.** Shown are the muscle arm defects of deletion alleles (as indicated by the *ok*, *gk*, or *tm* prefix) of the indicated MAP kinase component. **B.** Muscle-specific expression of SEK-3 (from the *him-4* promoter from construct pPRSAD936) is able to rescue *sek-3(ok1276)*’s suppression of *madd-3(tr186)*’s muscle arm defects. **C.** Muscle-specific over-expression of CEBP-1 (from the *him-4* promoter from construct pPRSAD961) phenocopies the *madd-3(tr186)* mutant phenotype from three distinct transgenic lines. For A-C, the presence of a wild type or *tr186* allele of MADD-3A is indicated with a respective (+) or (-) below the bars of the graph. In graphs A and C, counts for ventral left muscle 11 are shown, and in B, counts for dorsal right 15 is shown. In all graphs, an asterisk indicates a significant difference (p<0.01) compared to the data point indicated with a closed circle of the same color as the asterisks.

We tested the ability of the p38 MAPK components to suppress the muscle arm extension defects of *madd-3(tr186)*. We found that the deletion alleles of *sek-3*, *pmk-3*, *mak-2*, and *cebp-1* each suppress *madd-3(tr186)*’s muscle arm defects (Fig 6a). Muscle-specific expression of SEK-3 rescues *sek-3(ok1276)*’s suppression of *madd-3(tr186)*’s muscle arm extension defects (Fig 6b). Similarly, muscle-specific expression of CEBP-1 rescues *cebp-1(tm807)*’s suppression of the *madd-3* phenotype (Fig 6c, construct #2). These results suggest that this MAP kinase pathway functions cell-autonomously to regulate muscle arm extension. Consistent with the idea that MADD-3A negatively regulates this MAP kinase pathway, over-expression of the terminal component in the pathway, CEBP-1, induces muscle arm extension defects (Fig 6c, construct #1).

Surprisingly, *dlk-1(tm4024)* failed to suppress *madd-3*’s muscle arm defects despite being able to suppress the synthetic lethal phenotype of the *madd-3* mutant (Fig 6a). Our initial characterization of the suppressors of the *unc-54; madd-3* synthetic lethality revealed that the *dlk-1* ‘*tr*’ alleles failed to complement *pmk-3(tr347)*’s suppression of *madd-3(tr186)*’s muscle arm extension defects. Two examples of the *dlk-1* and *pmk-3* non-complementation are shown in Fig 6a. We repeated this test with a deletion allele of *dlk-1(tm4024)* and found that it too fails to complement *pmk-3*’s suppression of *madd-3(tr186)*’s muscle arm extension defects (Fig 6a). The *dlk-1/pmk-3* interaction is an example of non-allelic non-complementation and suggests that DLK-1 does indeed play a role in regulating muscle arm extension.

SEK-3’s paralog MKK-4 is also known to function downstream of DLK-1 [24,28]. We found that MKK-4 regulates muscle arm extension in an opposite fashion compared to SEK-3; a deletion allele of *mkk-4* not only confers muscle arm defects, but also enhances the muscle arm defects of the *madd-3(tr186)* mutant (Fig 6a). This suggests that a second MAP kinase pathway that includes MKK-4 may be acting in opposition to the SEK-3 MAP kinase pathway to regulate muscle arm extension (see below for more discussion).

Given that the genetic relationship between the p38 MAP kinase components and *madd-3* is similar to that of the p38 MAPK pathway and *rpm-1*, we asked whether an *rpm-1* null mutation might exhibit phenotypes similar to *madd-3(tr186)*. We found that *rpm-1* null mutants have fewer muscle arms than wild type controls and that the *rpm-1* null mutation fails to enhance the muscle arm extension defects of *madd-3(tr186)* (Fig 6a). These results suggest that MADD-3A and RPM-1 may function together to regulate muscle arm extension. We also tested whether the *pmk-3* mutant can suppress the muscle arm defects of the *rpm-1; madd-3* double mutant, and found that it could (Fig 6a). This provides additional evidence that MADD-3A may be working with RPM-1 to regulate muscle arm extension through the MAP kinase pathway.

### MADD-3A and the p38 MAP Kinase Pathway Interact to Regulate the Abundance of the EVA-1 Transmembrane Receptor

In *C*. *elegans* interneurons, RPM-1 and PMK-3 regulate AMPA receptor trafficking [28]. PMK-3 promotes receptor endocytosis, perhaps via the activation of the RAB-5 GTPase, while RPM-1 negatively regulates DLK-1, which consequently blocks PMK-3 activation. Inspired by this work, we asked whether MADD-3A and the p38 MAPK pathway might regulate the trafficking of the EVA-1 and UNC-40 receptors that direct muscle arm extension.

**Fig 7:**
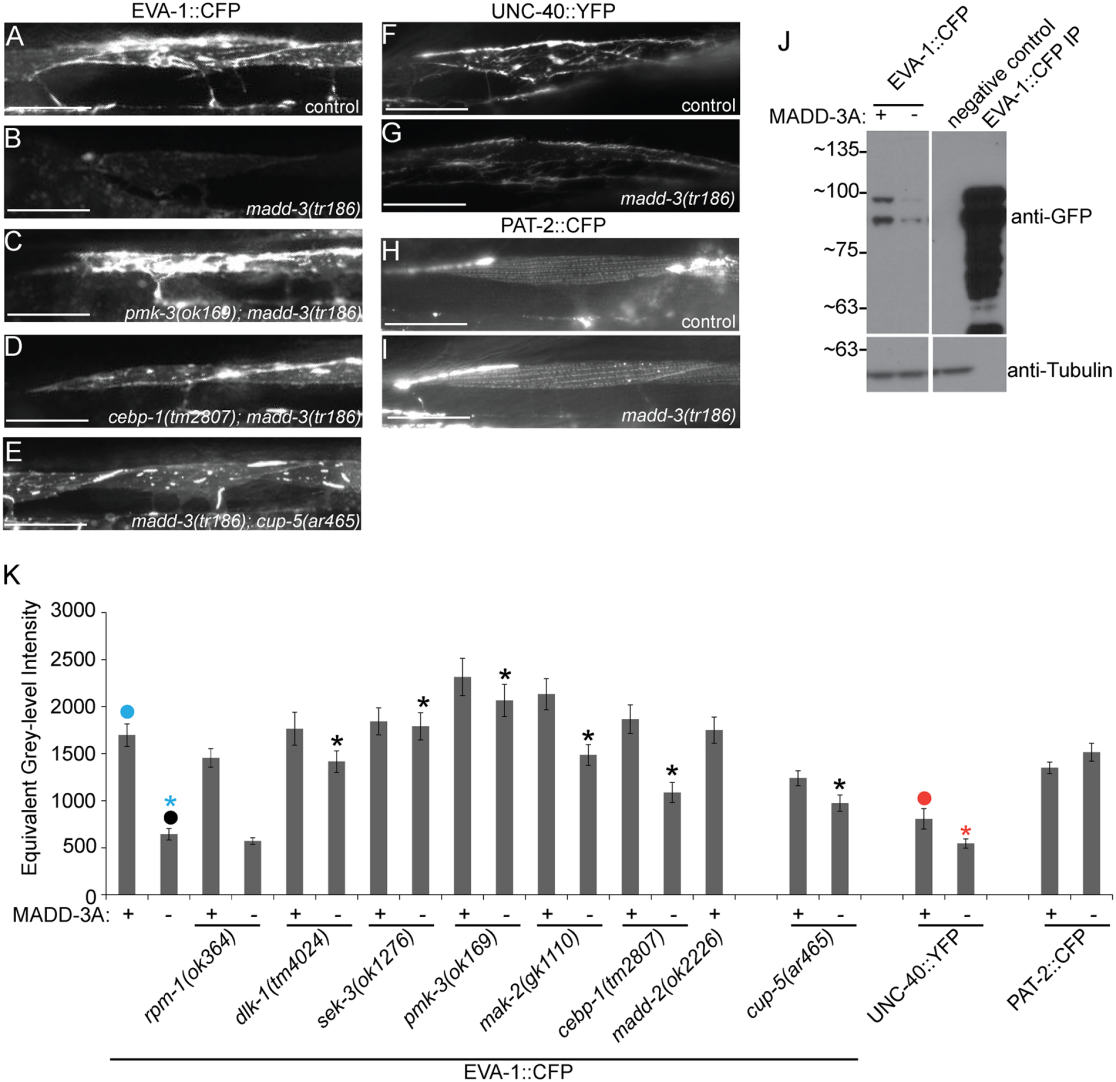
Mutations in p38 MAP kinase components and the mucolipin ortholog CUP-5 suppress the decrease in abundance of EVA-1 in *madd-3* mutants. **A-E.** Muscle cells from animals of the indicated genotype express EVA-1:: MYC::3XFLAG::CFP in muscles from the *trIs89* chromosomally integrated transgene. Each picture was taken with the same exposure time. **F-G.** Muscle cells from animals of the indicated genotype express UNC-40::YFP in muscles from the *trIs41* transgene. **H-I.** Muscle cells from animals of the indicated genotype express PAT-2::CFP in muscles from the *trIs72* integrated transgene. The scale bars in A-I represent 25μm. **J.** A western blot of whole worm lysate from a mixed stage population of the indicated genotype probed with an antibody against GFP (which recognizes CFP) (top) and an antibody against tubulin as a loading control (bottom). EVA-1::CFP is expressed from the integrated transgene *trIs89*. **K.** Quantification of fluorescent signal for the indicated transgene and genetic background. The transgenic protein whose abundance is being measured is indicated below the genotype at the bottom of the panel. An asterisk indicates a significant difference (*p*<0.01) compared to the data point indicated with a closed circle of the same color as the asterisks.

We examined a previously characterized functional CFP-tagged EVA-1 receptor that is specifically expressed in muscles [7]. In *madd-3(tr186)* mutants, there is a dramatic reduction of EVA-1::CFP (Fig 7a, 7b, 7j and 7k). This cannot be the result of altered EVA-1 splicing because the transgenic EVA-1 is encoded by an *eva-1* cDNA that lacks introns. There is also less muscle-expressed UNC-40::YFP in *madd-3(tr186)* mutants compared to controls (Fig 7f, 7g and 7k). By contrast, the abundance of muscle-expressed PAT-2::CFP remains unchanged in the *madd-3* mutant background, demonstrating that the *madd-3(tr186)* mutation is not a general suppressor of transgenic expression (Fig 7h, 7i and 7k). We found that null mutations in *dlk-1*, *sek-3*, *pmk-3*, *mak-2*, *and cebp-1* were able to suppress *madd-3(tr186)*’s repression of EVA-1::CFP abundance (Fig 7k).

We previously demonstrated that when CFP-tagged EVA-1 is expressed from muscles, it is able to recruit neuronally-expressed MADD-4::YFP to the muscle membrane [7]. We find that *madd-3(tr186)* dramatically reduces the ability of animals to recruit the neuronally-expressed MADD-4::YFP to muscle cells that express EVA-1::CFP (S4 Fig). We tested the ability of three null mutants of the MAP kinase pathway (*pmk-3*, *mak-2*, and *cebp-1*) to suppress this *madd-3* mutant phenotype, and found that each could restore the ability of the *madd-3(tr186)* mutants to recruit neuronally-expressed MADD-4::YFP to the muscles expressing EVA-1::CFP (S4 Fig). Hence, one reason why *madd-3* mutants have muscle arm extension defects may be because MADD-3A is required for the proper display of the EVA-1 and UNC-40 receptors that normally direct muscle arm extension towards sources of the MADD-4 attractive cue.

### MADD-3A Antagonizes EVA-1 Trafficking to the Lysosome

**Fig 8:**
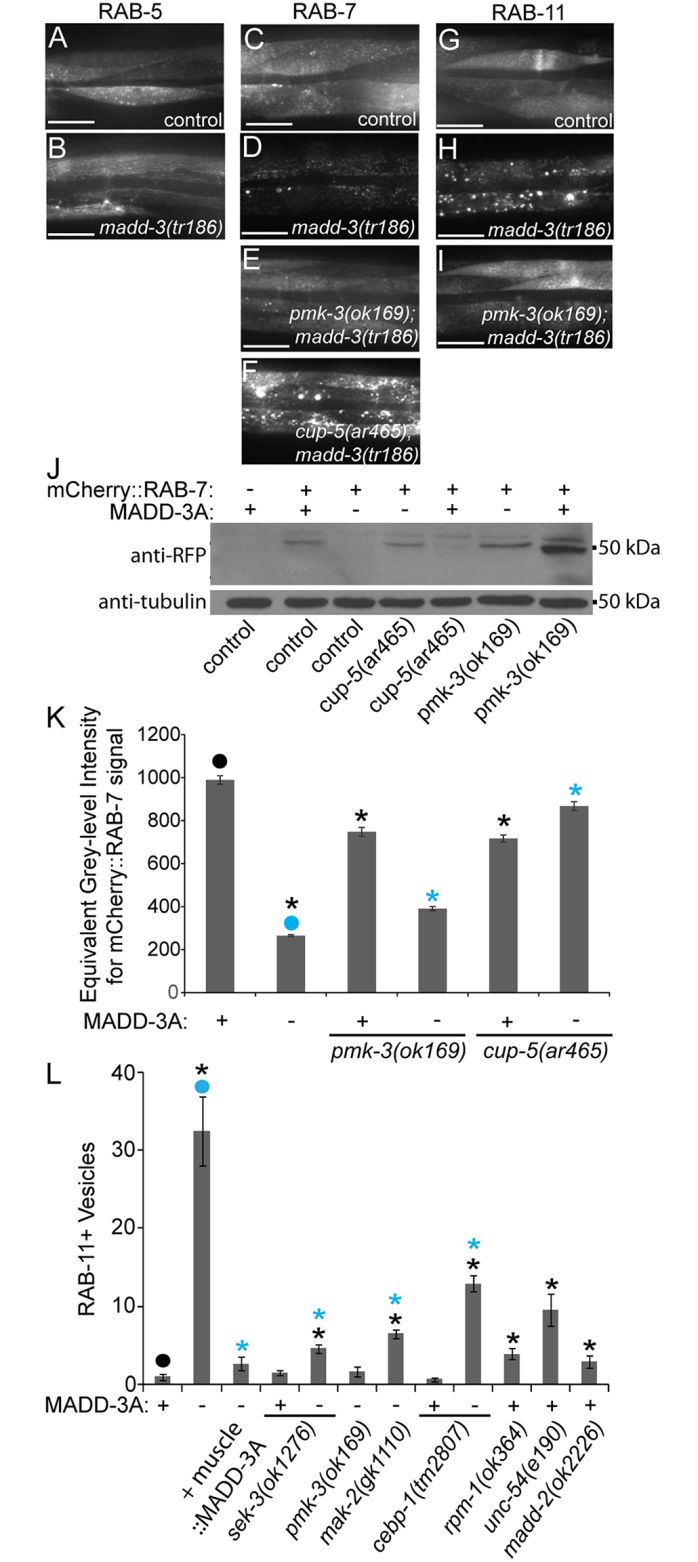
Mutations in p38 MAP kinase components and the mucolipin ortholog CUP-5 suppress the decrease in abundance of RAB-7 in *madd-3* mutants. **A-I.** The muscle cells of animals of the indicated genotype expressing the indicated vesicle marker. **A-B.** mCherry::RAB-5 is specifically expressed in muscles from an extrachromsomal array harboring the pPRSAD950 plasmid. **C-F.** mCherry::RAB-7 is specifically expressed in muscles from the *huIs89* integrated transgene. **G-I.** mCherry::RAB-11 is specifically expressed in muscles from the *huIs97* integrated transgene. All RAB reagents were kind gifts from Rik Korswagen. The scale bars in A-H represent 50 μm. **J.** Western blot analyses of whole worm lysate from a mixed stage population of the indicated genotype probed with anti-RFP antibodies, which recognize mCherry (top), and anti-tubulin antibodies (bottom) as a loading control. **K.** Quantification of the RAB-7 vesicle phenotype in the indicated genetic background. **L.** Quantification of the RAB-11 vesicle phenotype in the indicated genetic background. MADD-3A is specifically in muscles from the *myo-3* promoter from an extra-chromosomal array (from the pPRSAD499 plasmid). For K-L, the presence of a wild type or *tr186* allele of MADD-3A is indicated with a respective (+) or (-) below the bars of the graph. An asterisk indicates a significant difference (*p*<0.05) compared to the data point indicated with a closed circle of the same color as the asterisks.

We previously found evidence for EVA-1 packaging into endosomes [7]. We therefore investigated the possibility that EVA-1 abundance is diminished in *madd-3* mutants as a consequence of endosomal trafficking defects. In *madd-3* mutants, we examined the distribution of RAB-5, RAB-7, and RAB-11, which respectively mark the early, late, and recycling endosomes [30,31]. We observed no differences in the RAB-5 marker in *madd-3* mutants compared to control animals (Fig 8a and 8b), but saw an obvious decrease in the abundance of RAB-7 *in vivo* (Fig 8c, 8d and 8k) and on western blots (Fig 8j). We also found that the RAB-11 pattern is redistributed from a diffuse pattern to being punctate (Fig 8g, 8h and 8l). Disrupting the p38 MAP kinase pathway suppresses the *in vivo* changes observed with the RAB-7 and RAB-11 markers in the *madd-3(tr186)* mutants (Fig 8e and 8i–8l). Western analysis shows that mutations in *pmk-3* can restore RAB-7 levels in the *madd-3* mutant background (Fig 8j).

Given that EVA-1 and RAB-7 levels decrease in *madd-3* mutants, and that late endosomes fuse to the lysosome to degrade late endosome contents, we investigated the possibility that MADD-3A antagonizes endosomal trafficking of EVA-1 to the lysosome. To test this, we examined EVA-1 and RAB-7 abundance in *madd-3* mutants with disrupted CUP-5 function. CUP-5 is a mucolipin ortholog required for the proper function of the lysosome [32]. In *cup-5* mutants, late endosome fusion with lysosomes is disrupted and aberrant late endosome/lysosome hybrid organelles accumulate[32]. We reasoned that if EVA-1 and RAB-7 levels decrease in *madd-3* mutants for reasons other than increased trafficking to the lysosome, then disrupting CUP-5 will have little effect on EVA-1 and RAB-7 levels. We found that disrupting CUP-5 in *madd-3* mutants leads to an obvious increase in EVA-1 and RAB-7 levels (Figs 7e, 7k and 8f, 8j and 8k). Furthermore, the pattern of EVA-1 and RAB-7 is obviously vesicular in this background, suggesting that the *cup-5* mutation blocks the trafficking of EVA-1-containing vesicles to the lysosome (Figs 7e and 8f). These data are consistent with the idea that MADD-3A normally antagonizes EVA-1 trafficking to the lysosome via RAB-7-marked endosomes.

## Discussion

We identified *madd-3(tr186)* in a forward genetic screen for mutants with disrupted muscle arm extension. Other genes identified in our screens include the MADD-4 guidance cue, the UNC-40 and EVA-1 co-receptors, the MADD-2 adaptor protein, cytoskeletal components (actin) and their regulators (UNC-60B, UNC-73, LEV-11, GEX-2, GEX-3 and WVE-1), components important for muscle structure (PAT-2, PAT-3, UNC-95, UNC-97, and UNC-98), sarcomere function (UNC-54, UNC-15), and extracellular matrix components (UNC-52, LAM-1 and LAM-2) [1,2,33]. Hypotheses about the mechanism by which each of these proteins regulate muscle arm extension could be readily derived from the type of protein they encode, their expression and localization pattern, and their pattern of genetic interactions with one another. By contrast, how MADD-3 regulates muscle arm extension remained enigmatic until we identified genetic suppressors of *madd-3*’s synthetic lethal interaction with *unc-54* through a second genetic screen.

**Fig 9:**
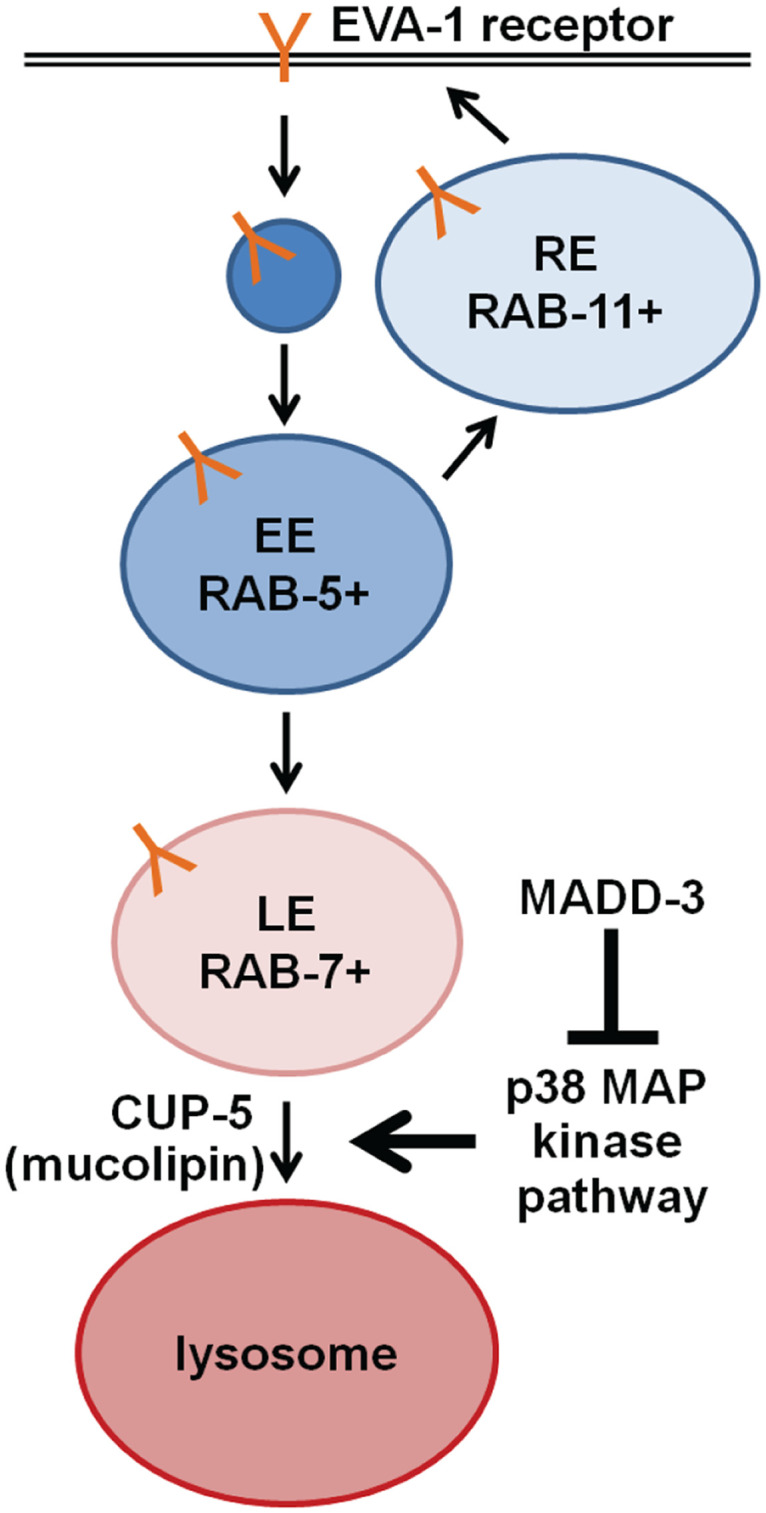
A model of the relationship between MADD-3, EVA-1 and the p38 MAP kinase pathway. The EVA-1 receptor, which is required to guide muscle arm extension towards the MADD-4 guidance cue, is indicated in orange. RE, recycling endosome; EE, early endosome; LE, late endosome.

We found that loss-of-function mutations in a p38 MAP kinase pathway, which includes DLK-1, SEK-3, PMK-3, MAK-2, and CEBP-1, suppress each of the *madd-3* mutant phenotypes (either as homozygotes or as trans-heterozygotes). These phenotypes include muscle arm extension defects, reduced receptor levels, changes in endocytic markers, and synthetic lethality with mutants of UNC-54 myosin heavy chain B. We also found that CEBP-1 over-expression phenocopies the muscle arm extension defects of *madd-3* mutants. Together, these observations suggest that MADD-3A negatively regulates the p38 MAP kinase pathway (Fig 9). How MADD-3A might negatively regulate the p38 pathway is not yet clear, but given that loss-of-function mutations in the top-most components of the pathway are sufficient for suppression, MADD-3A is unlikely to act on components downstream in the pathway.

One mechanism by which MADD-3A might regulate the p38 MAPK pathway is via the regulation of the ubiquitin ligase RPM-1. Of all of the mutants with which we have investigated *madd-3* interactions, the *rpm-1* null allele is the only one that both confers muscle arm defects and fails to enhance the muscle arm defects of *madd-3(tr186)*. In addition, both MADD-3A and RPM-1 appear to negatively regulate a p38 MAPK pathway. These observations suggest that MADD-3A and RPM-1 may work close together. Given that RPM-1 targets DLK-1 for degradation [24,28], one potential role for MADD-3A might be to prime DLK-1 for RPM-1 recognition via DLK-1 phosphorylation, or regulate RPM-1 itself via phosphorylation. However, MADD-3A and RPM-1 are unlikely to function as an obligatory pair because *rpm-1* mutants do not have obvious defects in EVA-1 receptor display. Hence, if MADD-3 interacts with RPM-1 to regulate muscle arm extension, this branch of the pathway is doing so independent of the regulation of EVA-1 levels.

The interactions between MADD-3 and the p38 map kinase pathway are complex. First, while homozygous null mutants of SEK-3, PMK-3, MAK-2, and CEBP-1 can suppress each of the *madd-3* mutant phenotypes that we examined, the homozygous *dlk-1* null mutation could suppress only a subset of these phenotypes. Mutant heterozygotes of *dlk-1* are able to suppress *madd-3*’s muscle arm extension defects in combination with heterozygous *pmk-3* mutations. In neurons, the p38 MAPK pathway that regulates presynaptic development includes DLK-1, but instead of the SEK-3 MAPKK, the pathway employs the MKK-4 MAPKK [24,25]. Surprisingly, we found that the *mkk-4* null mutant has the opposite effect compared to the *sek-3* null mutant and enhances the muscle arm extension defects of the *madd-3* mutant. This suggests that DLK-1 may regulate muscle arm extension via two MAPK pathways; one that positively regulates muscle arm extension (and includes MKK-4) and one that negatively regulates it (and includes SEK-3). This duality may be the reason behind *dlk-1*’s complex genetic interaction with *madd-3* in regulating muscle arm extension. Second, *cebp-1* loss-of-function can suppress *madd-3*’s muscle arm extension defects and its reduction of EVA-1 abundance. Consistent with these results, over-expression of CEBP-1 induces muscle arm extension defects. However, we found that the over-expression of CEBP-1 fails to reduce EVA-1::CFP levels, suggesting that CEBP-1’s regulation of muscle arm extension is complex.

Discovering that defects in the p38 MAP kinase pathway suppress the phenotypes of *madd-3* mutants led to the insight that MADD-3A is required for maintaining the abundance of the EVA-1 and UNC-40 receptors on the muscle plasma membrane. EVA-1’s role in directing muscle arm extension is entirely dependent on UNC-40, but UNC-40 (and its MADD-2 adaptor) has EVA-1-independent roles in this process [7]. Given that MADD-3A regulates the display of both receptors, it is not surprising that *madd-3(tr186)* enhances the defects of null mutants in both the MADD-4-EVA-1 branch (i.e. *madd-4* and *eva-1* null mutants), as well as the EVA-1-independent UNC-40 branch (i.e. *madd-2* null mutants). Hence, the decrease in EVA-1 and UNC-40 abundance is likely to contribute to the muscle arm extension defects of *madd-3* mutants.

The examination of endosomal markers and the effects of disrupting late-endosome to lysosome fusion events suggests a model for MADD-3A and the p38 MAP Kinase pathway function in muscle arm extension (Fig 9). In this model, MADD-3A negatively regulates the p38 pathway, which normally promotes late endosome formation and/or fusion with the lysosome in wild type animals. EVA-1 may normally cycle through early endosomes and recycling endosomes [7]. Animals lacking MADD-3A increase p38 MAP Kinase activity, which in turn promotes excessive trafficking of EVA-1-containing endosomes to the lysosome. This results in a paucity of the late endosomal marker RAB-7, a decrease of EVA-1 at the plasma membrane and a consequent defect in muscle arm extension. Why the RAB-11 recycling endosome marker changes from diffuse to vesicular in *madd-3* mutants is unclear, but may be compensatory homeostatic mechanism. Whether an endosomal sorting defect is also the root cause of the synthetic lethality of *madd-3* mutants with *unc-54* is unknown. Regardless, our work provides the first evidence that links the LAMMER and p38 MAP kinase families that are both conserved throughout eukaryotes and provides a foundation to further investigate non-splicing activities of the LAMMER kinases in other systems.

## Materials and Methods

### Nematode Strains and Transgenics

All nematode strains were cultured at 20°C on OP50 *E*. *coli*-seeded agar plates using standard protocols [33]. All mutants were obtained from the *Caenorhabditis* Genetics Center, except those designated with an RP or *tr* prefix, which were generated in our lab.

The following extra chromosomal arrays were constructed via microinjection (for the indicated experiment: MADD-3A expression from endogenous promoter for both localization and rescue–[pPRSAD539 (*madd-3ap*::YFP::MADD-3A)(5ng/μl); pRF4 (*rol-6(su1006)*(100 ng/μl)]; MADD-3A muscle expression for rescue–[pPRAVS499 (*myo*-3p::MADD-3A::CFP)(5ng/μl); pPRGS382(*myo-2p*::mCherry)(2ng/μl); pKs (100ng/μl)]; MADD-3A distal muscle expression for localization and rescue–[pPRSAD426 (*him-4p*::MADD-3A::CFP)(5ng/ul); pRF4 (*rol-6(su1006)*(100ng/μl)]; MADD-3A-kinase dead distal muscle expression for localization and rescue–[pPRSAD534 (*him-4p*::MADD-3A(K580R)::CFP)(5ng/μl); pRF4 (*rol-6(su1006)*(100ng/μl)]; MADD-3A-kinase dead distal muscle expression for localization and rescue–[pPRSAD535 (*him-4p*::MADD-3A(DFG712AFG)(5ng/μl); pRF4 (*rol-6(su1006)* (100ng/μl)]; MADD-3 Kinase Domain muscle expression for localization and rescue–[pPRSAD427 (*him-4p*::MADD-3(kinase domain)::CFP)(5ng/μl); pRF4 (*rol-6(su1006)*(100ng/μl)]; MADD-3A expression in the nervous system–[pPRSAD937 (*F23B3*.*3p*::MADD-3A::CFP)(5ng/μl); pPRGS382(*myo-2p*::mCherry) (2ng/μl); pKs (100ng/μl)]; RAB-5 expression in the muscle–[pPRSAD950 (*myo-3p*::mCherry::RAB-5)(1ng/μl); pPRGS382(*myo-2p*::mCherry) (2ng/μl); pKs (100ng/μl)]; RAB-5(GTP) in the muscle–[pPRSAD951 (*myo-3p*::mCherry::RAB-5(Q78L)(1ng/μl); pPRGS382(*myo-2p*::mCherry) (2ng/μl); pKs (100ng/μl)]; RAB-5(GDP) in the muscle–[pPRSAD952 (*myo*-3p::mCherry::RAB-5(S33N)(1ng/μl); pPRGS382(*myo-2p*::mCherry) (2ng/μl); pKs (100ng/μl)]; Visualization of sarcomeres–*raEx267*[*mlc-3p*::MCL-3::GFP, *pha-1(+)*, pRF4 (*rol-6(su1006*) (100ng/μl)].

The following chromosomally-integrated transgenic arrays were generated in our lab using standard techniques: *trIs30* [pPRRF138.2 (*him-4p*::MB::YFP)(10 ng/μl); pPRZL44 (*hmr-1bp*::DsRed2)(80 ng/μl); pPR2.1 (*unc-129nsp*::DsRed2)(40 ng/μl)] I; *trIs41* [pPRKC294 (*him-4p*::UNC-40::YFP)(125 ng/μl); pRF4 (*rol-6(su1006*)(100 ng/μl)] II; *trIs42; trIs50; trIs66* [pPRFS628 (*unc-119p*::MADD-4B::MYC::3XFLAG::YFP)(50 ng/μl); pPRGS382 (*myo-2p*::mCherry)(5 ng/μl)] X; *trIs72; trIs89* [pPRKC793 (*him-4p*::EVA-1::MYC::3XFLAG::CFP)(50 ng/μl); pPRGS382 (*myo-2p*::mCherry)(2 ng/μl)].

The following chromosomally-integrated transgenes were gifts from Rik Korswagen: *huIs89* [*myo-3p*::mCherry::RAB-7]; *huIs97* [*myo-3p*::mCherry::RAB-11].

### A Genetic Screen for New Mutants with Defective Muscle Arm Extension

A genetic screen for *C*. *elegans m*uscle *a*rm *d*evelopment *d*efective (Madd) mutants was performed by incubating a mixed stage population of RP112 *trIs25 [pPRRF138*.*2(him-4p*::*MB*::*YFP)*, *pPRZL47(F25B3*.*3p*::*DsRed2)*, *pRF4(rol-6(su1006)]; rrf-3(pk1426)* animals in 50 mM ethyl methanesulfonate (EMS) for 4 hr. pRF4, a plasmid that encodes a dominant mutation in *rol-6(su1006)*, which induces a rolling phenotype, was used to facilitate the observation of muscle arm extension in the living animals [34,35]. *rrf-3(pK1426)* was used to decrease the visual noise imparted by the *pPRRF138*.*2* transgene [2]. After 4 hours the mutagenized population was rinsed, fed for 24 hours and then the embryos were harvested. The resulting F1 population of synchronized L1s were then grown on 6 cm plates (approximately 3000 per plate). A COPAS Biosort (Union Biometrica, Inc) was used to place three L3-stage F1 worms per well in 12-well plates seeded with OP50 *E*.*coli* and the F2 progeny were screened four days later for mutants with muscle arm extension defects using a Leica MZFLIII epiflourescence dissection microscope with a 2× objective. m*add-3(tr186)* was mapped using the snip-SNP molecular mapping technique as previously described [2] [36].

### Phenotypic Analyses and Photomicrosopy

Worms were anaesthetized in 1mg/mL levamisole (Sigma) in M9 solution and mounted on a 1.5% agarose pad to immobilize them for photomicrosopy. We used a Leica DMRA2 HC microscope with standard Leica filter sets for GFP, YFP, CFP and DsRed epiflourescense to take all pictures.

All muscle arm counts were performed in the background of chromosomally integrated transgenic array *trIs30* I (described above) [2], which expresses membrane-anchored YFP from the *him-4* promoter in select distal body wall muscles.

RAB-11 vesicle counts were performed in the background of *huIs97*. Strains were visualized and images captured using Leica DMRA2 HC with an RFP filter at 20X and 40X. From the 20X images, the number of RAB-11+ vesicles in the ventral anterior muscles (VL9, 10, 11, 12, 13 and 14) were counted for 10 animals.

Signal from the EVA-1::CFP, UNC-40::YFP, PAT-2::CFP, and mCherry::RAB-7 fluorescent strains was quantified by capturing images at the same exposure using a Leica DMRA HC with appropriate filter sets at 40X and 20X. Signal was quantified using OpenLab software to measure pixel intensity in a standard Region of Interest (R.O.I.). At least 25 different animals were analyzed per sample. Pixel intensity counts captured in different months were normalized relative to a *madd-3(tr186)* control.

All statistical analyses were performed using a two-tailed, two sample equal variance Student’s T-Test.

### A Forward Genetic Screen for Mutants that Suppress the *madd-3; unc-54* Synthetic Lethality

A genetic screen for *madd-3 suppressor* mutants was performed by incubating a mixed stage population of RP2361 *madd-3(tr186); unc-54(e190)*, *trIs30*; Ex[*pPD5*.*41*(*unc-54p*::UNC-54; *pPD133*.*51* (*myo-3p*::CFP)] in 50 mM ethyl methanesulfonate (EMS) for 4 hours. Resulting F1s were synchronized as L1s and grown on 6 cm plates (with approximately 3000 animals per plate). The F1 plates were divided into pools and each pool was synchronized separately; the resulting F2 pools plated on the center of 10cm plates (a maximum of 6000 per plate). When the F2 worms reached L4/young adult (approximately 4 days) the center of each plate was chunked onto another 6cm plate; this maximized the detection of RP2361-derived animals that no longer had the extra-chromosomal array (as detected with a Leica MZFLIII epiflourescence dissection microscope with a 2× objective) and retained the *unc-54(e190)* paralyzed phenotype. Resulting animals of this phenotype were considered candidate *madd-3(tr186)* suppressors. Ultimately, only one strain derived from each F2 pool was characterized in detail to ensure that candidate suppressors were independently isolated (i.e. not siblings of one another).

### Whole Genome Sequencing of the ma*dd-3; unc-54* Suppressors

The genomes of the *madd-3(tr186)* suppressors were sequenced as previously described [29]. Briefly, a DNA library was generated from each strain using a Nextera DNA Sample Preparation Kit (Illumina). We sequenced the strains in multiplex fashion in two batches for historical reasons with paired end reads using version 4 reagents and flow cells using the Donnelly Sequencing Centre’s (Toronto, Ontario) Illumina HiSeq2500. Data was processed by Andrew Fraser’s group at the Donnelly Centre for Cellular and Biomolecular Research. The quality of the raw sequence reads was first assessed using fastqc. The reads were then mapped to the *C*. *elegans* reference genome (WS220) using a combination of GATK and Picard software. HaplotypeCaller was then used to identify variants in the suppressor genomes, and Annovar was used to annotate the raw variants with functional information. The Annovar output was converted into an excel file that we manually inspected (see S1 Dataset). We removed synonymous mutations from the file, then sorted the data to show variants that caused unique homozygous mutations in each genome. We then identified commonly mutated genes that had distinct mutations in distinct suppressor strains.

## Supporting Information

S1 Table An estimation of the probability that any single collection of 25 mutant strains will have the same gene mutated (with a mutation that alters protein coding sequence) in a given number of the 25 strains by random chance alone. The calculations of the probabilities shown in S1 Table were performed by Prof. Fritz Roth (University of Toronto) and are based on the following information: The predicted number of protein-coding genes in the *C*. *elegans* genome = 20514 (WormBase Release WS229); the average coding length of a given gene = 1248 bp (total coding sequence in the genome = 25,601,472 bp [37]; the average number of genes that have mutations that alter the protein coding sequence per mutant strain based on an EMS concentration of 50 mM (which is what we use); the number of mutant strains that we sequence is 25.
(PDF)

S1 Fig The mapping and identification of the *madd-3* gene responsible for the *tr186* phenotype. *tr186* was initially identified as being sex-linked (as indicated with the red bar on the X chromosome) through a variety of crosses. snip-SNP mapping was performed as previously described (see methods). Briefly, we crossed the N2-derived *tr186*-harboring strain (whose DNA is represented by a red line) into the CB4856 strain (that contains small nucleotide polymorphisms that create or destroy restriction endonuclease sites relative to the N2 genome and whose DNA is represented by a blue line). We identified recombinants with breakpoints that indicated that the *tr186* mutation resides in a 135 kb region on chromosome X that contains 26 protein-coding genes (schematized with arrows). Sequencing of a candidate gene for which a systematic expression analysis indicated muscle expression (E02H4.3- brown arrow) revealed the *tr186* mutation.
(TIF)

S2 Fig A comparison of the kinase domains of select LAMMER kinase family members. A multiple sequence alignment of the kinase domains of members of the LAMMER kinase family. The eponymous EHLAMMERILG motif is underlined in red. Underlined in blue are two additional motifs that distinguish LAMMER kinases [8]. Black indicates identical residues; grey indicates similar residues. A red asterisk marks an invariant lysine residue in protein kinase subdomain II found in all kinases that is required for phosphate transfer [38], which we have mutated to K580R in the pPRSAD534 construct (see Fig 3). Blue asterisk marks an aspartic acid in the DFG motif of subdomain VII that is involved in cation binding and orientation of the ATP gamma phosphate for phosphate transfer [39], which we have mutated to DFG712AFG in the pPRSAD535 construct (see Fig 3).
(TIF)

S3 Fig A schematic of the genetic screen designed to isolate *madd-3* suppressors. *unc-54(e190); madd-3(tr186)* double mutants are sub-viable without an extra-chromosomal array that expresses either MADD-3 or UNC-54 in muscles. Here, UNC-54(+) is expressed specifically in muscles from an extra-chromosomal array (that harbors the *pPD5*.*41* plasmid (a wild type UNC-54 genomic construct) and the *pPD133*.*51* (*myo-3p*::CFP) co-injection marker that drives CFP expression in muscles. *pPD5*.*41* and *pPD133*.*51* are gifts from Andrew Fire. After the mutagenesis of parents, uncoordinated F1 mutants that are viable and do not fluoresce (and therefore lack the extra-chromosomal array- indicated by the yellow box) were isolated and characterized further.
(TIF)

S4 Fig MADD-3A is required for muscle-expressed EVA-1 to recruit neuronally-expressed MADD-4 to the membrane of muscles. Each row focuses on a single ventral muscle cell whose genetic background is indicated on the right. In the left column, only the CFP channel is shown; in the second column, only the YFP channel is shown; the merge is shown in the third row. Animals express EVA-1::CFP from the *trIs89* chromosomally-integrated transgene and MADD-4 from the *trIs66* chromosomally-integrated transgene. Note that the reduction of MADD-4::YFP recruitment by EVA-1 that is seen in the *madd-3(tr186)* background is suppressed by mutations in MAP kinase components. The scale bar represents 25 μm for all images.
(TIF)

S1 Dataset Whole genome sequence of 25 *madd-3(tr186)* suppressor mutants. The 25 mutants were sequences in two batches and the resulting homozygous mutations in each strain are reported in two files in two corresponding excel tabs. Only protein changing mutations are reported. The alleles are indicated in the top row. See methods for more details.
(XLSX)
