# Supplementary material for: The MADD-3 LAMMER Kinase Interacts with a p38 MAP Kinase Pathway to Regulate the Display of the EVA-1 Guidance Receptor in Caenorhabditis elegans
Source: PLoS Genet. 2016 Apr 28;12(4):e1006010. doi: 10.1371/journal.pgen.1006010 (PMC4849719; doi:10.1371/journal.pgen.1006010)
Supplement: S1 Table — The calculations of the probabilities shown in S1 Table were performed by Prof. Fritz Roth (University of Toronto) and are based on the following information: The predicted number of protein-coding genes in the C. elegans genome = 20514 (WormBase Release WS229); the average coding length of a given gene = 1248 bp (total coding sequence in the genome = 25,601,472 bp [37]; the average number of genes that have mutations that alter the protein coding sequence per mutant strain based on an EMS concentration of 50 mM (which is what we use); the number of mutant strains that we sequence is 25. (PDF) [file pgen.1006010.s001.pdf]

The Number of Times a Given Gene is Found Mutated in a Collection of 10 Resistant Mutants (X)	The Chance of Finding Any Given Gene to be Mutated in X Number of Strains by Random Chance (nominal P-value)[a]	The Chance of Finding Any Gene to be Mutated in X Number of Strains by Random Chance (Bonferroni-adjusted P-value)[b]
0	1	1
1	0.09	1
2	0.004	1
3	1.4E-04	0.9
4	3.5E-06	0.07
5	6.7E-08	0.001
6	1.1E-09	2.2E-05
7	1.5E-11	3.1E-07
8	1.8E-13	3.7E-09
9	0	0
10	0	0

Supplemental Table 1. An estimation of the probability that any single collection of 25 mutant strains will have the same gene mutated (with a mutation that alters protein coding sequence) in a given number of the 25 strains by random chance alone.

The calculations are based on the following information:
The predicted number of protein-coding genes in the *C. elegans* genome = 20514(WormBase Release WS229).
The average coding length of a given gene= 1248 bp (total coding sequence in the genome=25,601,472 bp (Spieth and Lawson, 2006).
The average number of genes that have mutations that alter the protein coding sequence per mutant strain based on an EMS concentration of 50 mM (which is what we use)= 80 (Flibotte et al., 2010).
The number of mutant strains that we sequence is 25.

[a]These values show a relatively liberal estimation of the chances of finding the same gene mutated in X number of strains based on a Poisson distribution. Based on random chance alone, any given gene will be mutated in two of the 25 mutant strains in one out of ~224 sets of 25 mutants for example.

[b]These values show a relatively conservative estimation of the chances of finding the same gene mutated in X number of strains based on a Bonferroni-adjusted calculation using the Sidak method.

Literature Cited

Flibotte, S., Edgley, M. L., Chaudhry, I., Taylor, J., Neil, S. E., Rogula, A., Zapf, R., Hirst, M., Butterfield, Y., Jones, S. J. et al. (2010). Whole-genome profiling of mutagenesis in Caenorhabditis elegans. *Genetics* 185, 431-41.

Spieth, J. and Lawson, D. (2006). Overview of gene structure. *WormBook*, 1-10.
