# Supplementary figures and images for: The MADD-3 LAMMER Kinase Interacts with a p38 MAP Kinase Pathway to Regulate the Display of the EVA-1 Guidance Receptor in Caenorhabditis elegans
Source: PLoS Genet. 2016 Apr 28;12(4):e1006010. doi: 10.1371/journal.pgen.1006010 (PMC4849719; doi:10.1371/journal.pgen.1006010)

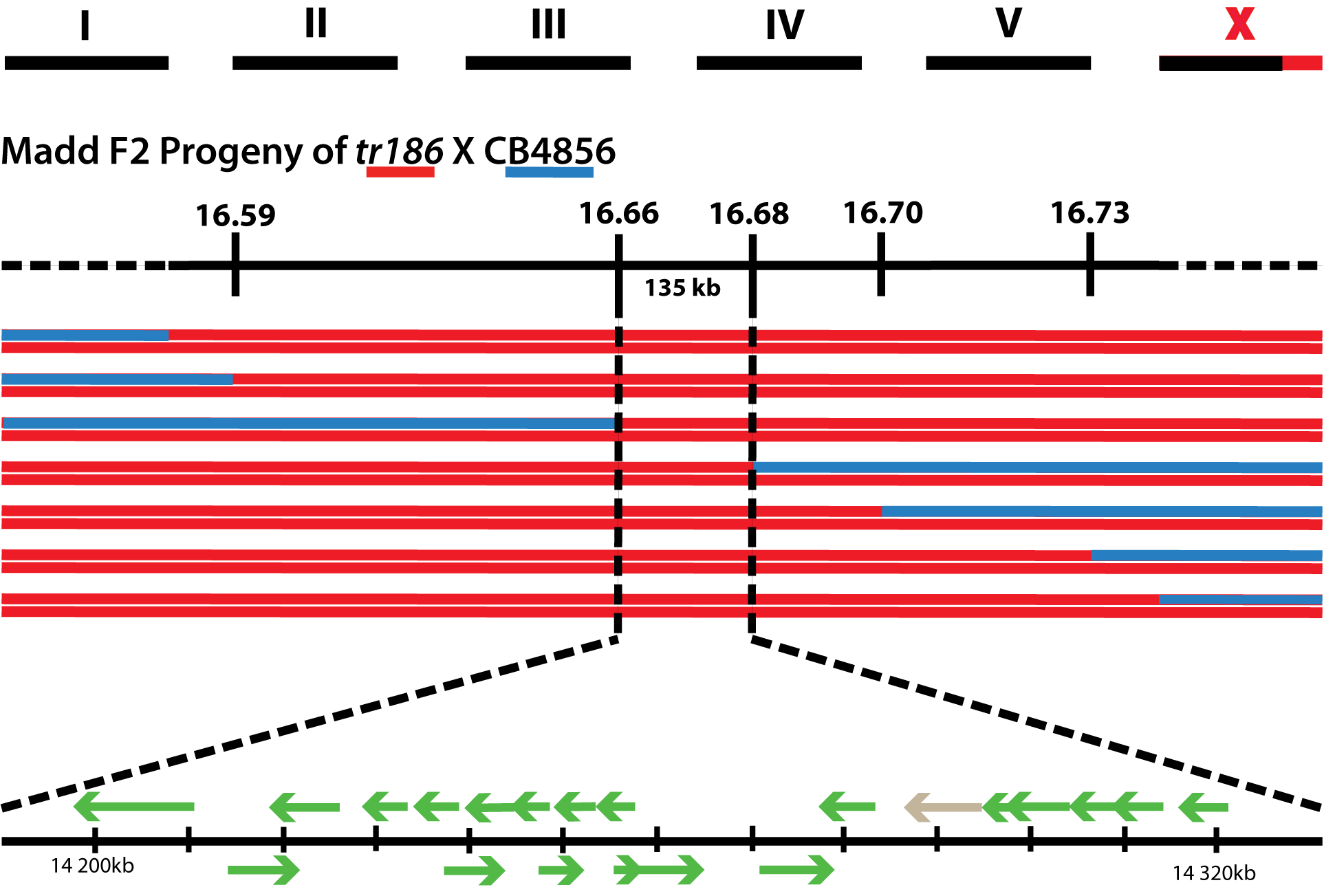

Supplement: S1 Fig — tr186 was initially identified as being sex-linked (as indicated with the red bar on the X chromosome) through a variety of crosses. snip-SNP mapping was performed as previously described (see methods). Briefly, we crossed the N2-derived tr186-harboring strain (whose DNA is represented by a red line) into the CB4856 strain (that contains small nucleotide polymorphisms that create or destroy restriction endonuclease sites relative to the N2 genome and whose DNA is represented by a blue line). We identified recombinants with breakpoints that indicated that the tr186 mutation resides in a 135 kb region on chromosome X that contains 26 protein-coding genes (schematized with arrows). Sequencing of a candidate gene for which a systematic expression analysis indicated muscle expression (E02H4.3- brown arrow) revealed the tr186 mutation. (TIF) [file pgen.1006010.s002.tif]

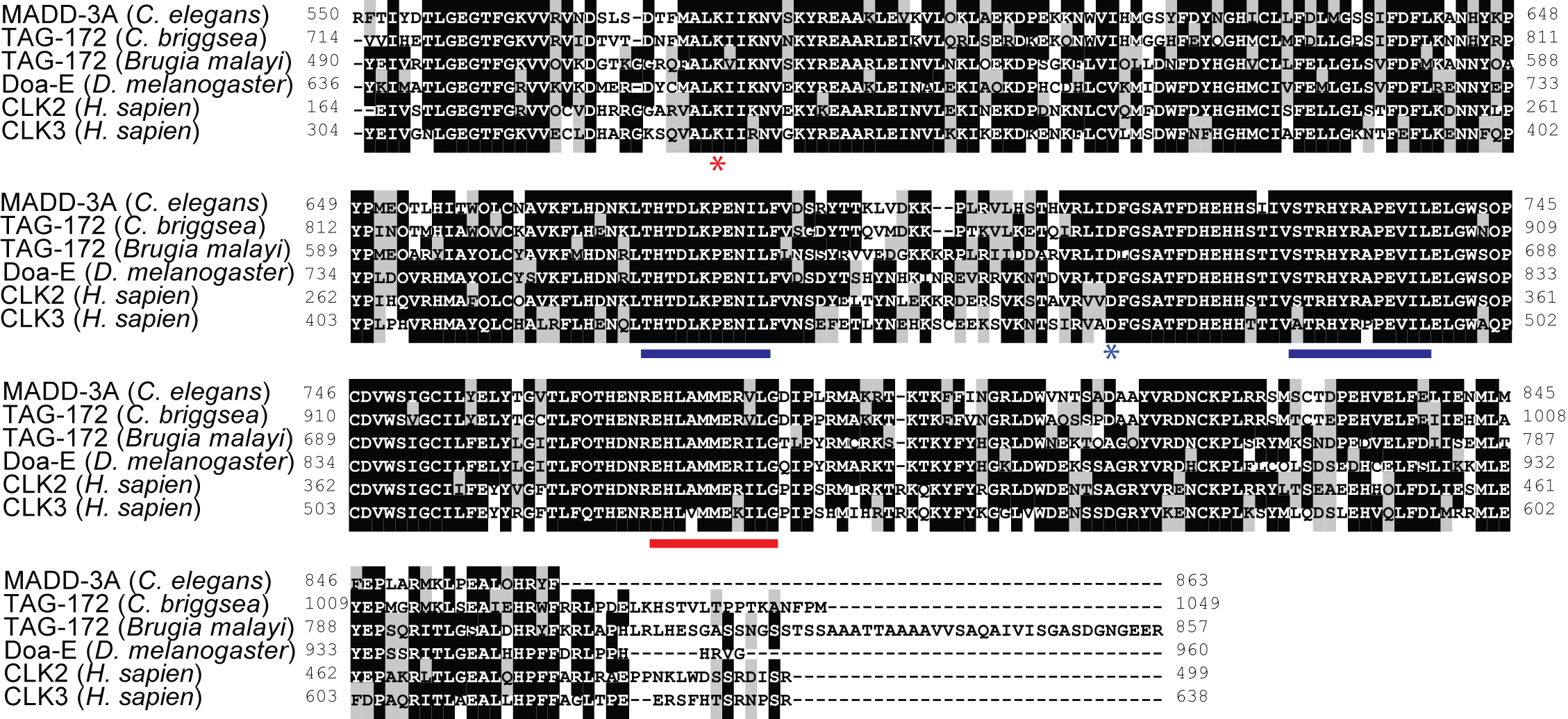

Supplement: S2 Fig — A multiple sequence alignment of the kinase domains of members of the LAMMER kinase family. The eponymous EHLAMMERILG motif is underlined in red. Underlined in blue are two additional motifs that distinguish LAMMER kinases [8]. Black indicates identical residues; grey indicates similar residues. A red asterisk marks an invariant lysine residue in protein kinase subdomain II found in all kinases that is required for phosphate transfer [38], which we have mutated to K580R in the pPRSAD534 construct (see Fig 3). Blue asterisk marks an aspartic acid in the DFG motif of subdomain VII that is involved in cation binding and orientation of the ATP gamma phosphate for phosphate transfer [39], which we have mutated to DFG712AFG in the pPRSAD535 construct (see Fig 3). (TIF) [file pgen.1006010.s003.tif]

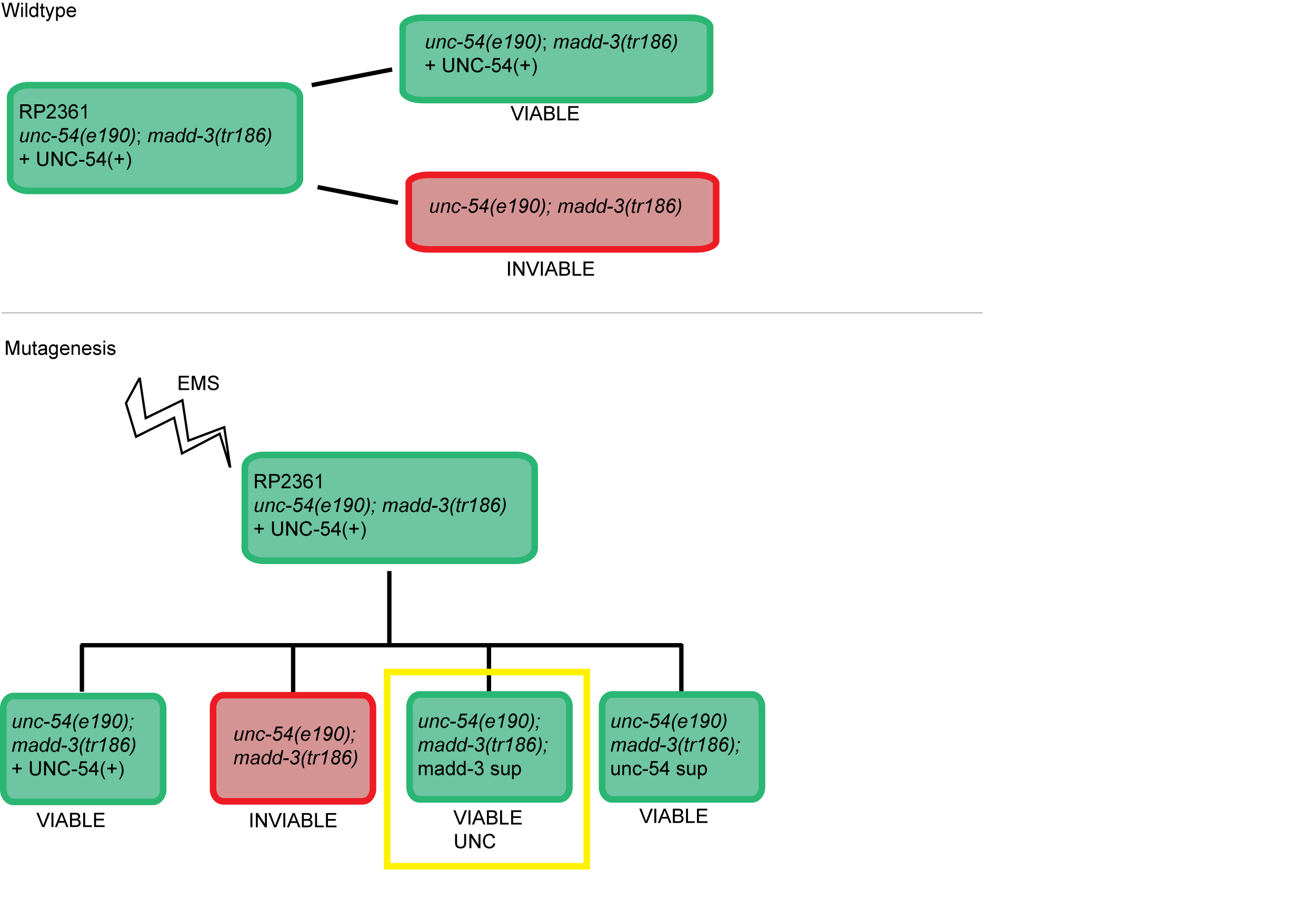

Supplement: S3 Fig — unc-54(e190); madd-3(tr186) double mutants are sub-viable without an extra-chromosomal array that expresses either MADD-3 or UNC-54 in muscles. Here, UNC-54(+) is expressed specifically in muscles from an extra-chromosomal array (that harbors the pPD5.41 plasmid (a wild type UNC-54 genomic construct) and the pPD133.51 (myo-3p::CFP) co-injection marker that drives CFP expression in muscles. pPD5.41 and pPD133.51 are gifts from Andrew Fire. After the mutagenesis of parents, uncoordinated F1 mutants that are viable and do not fluoresce (and therefore lack the extra-chromosomal array- indicated by the yellow box) were isolated and characterized further. (TIF) [file pgen.1006010.s004.tif]

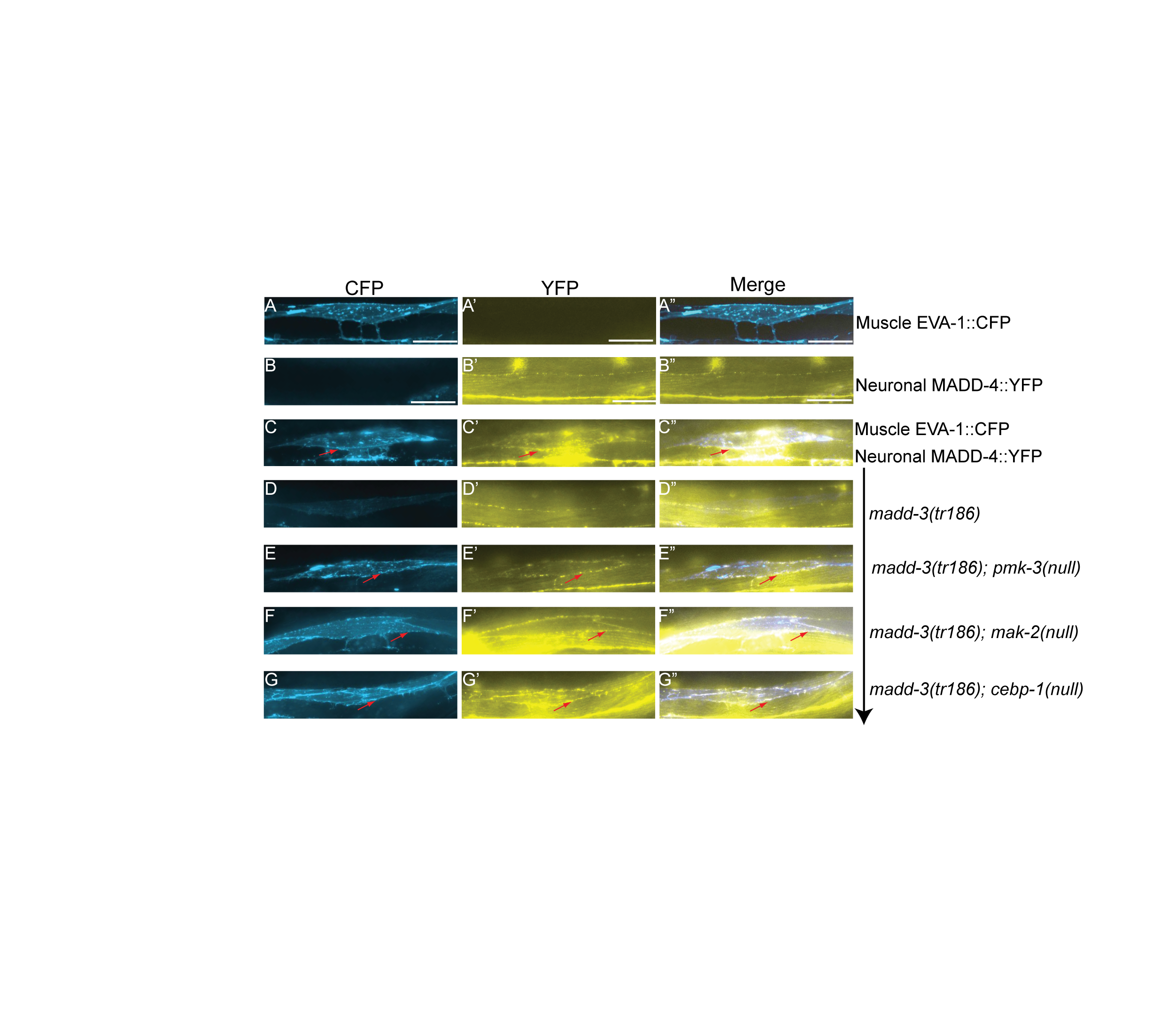

Supplement: S4 Fig — Each row focuses on a single ventral muscle cell whose genetic background is indicated on the right. In the left column, only the CFP channel is shown; in the second column, only the YFP channel is shown; the merge is shown in the third row. Animals express EVA-1::CFP from the trIs89 chromosomally-integrated transgene and MADD-4 from the trIs66 chromosomally-integrated transgene. Note that the reduction of MADD-4::YFP recruitment by EVA-1 that is seen in the madd-3(tr186) background is suppressed by mutations in MAP kinase components. The scale bar represents 25 μm for all images. (TIF) [file pgen.1006010.s005.tif]
